# Itaconate and obesity-related hormones promote tumor progression – new insights on metabolic dysfunction in early-onset colon cancer

**DOI:** 10.3389/fimmu.2025.1572985

**Published:** 2025-06-09

**Authors:** Katharina M. Scheurlen, Jacob Hallion, Dylan L. Snook, Anne MacLeod, Robert J. Beal, Mary A. Parks, Andrew B. Littlefield, Eliah Hiken, Adrian T. Billeter, Jeannette Bensen, Jeremy T. Gaskins, Julia Chariker, Eric C. Rouchka, Susan Galandiuk

**Affiliations:** ^1^ Digestive Surgery Research Laboratory, Price Institute of Surgical Research, The Hiram C. Polk, Jr., MD Department of Surgery, University of Louisville, Louisville, KY, United States; ^2^ Department of Visceral Surgery, Clarunis-University Digestive Healthcare Center, St. Claraspital and University Hospital Basel, Basel, Switzerland; ^3^ Lineberger Comprehensive Cancer Center, University of North Carolina at Chapel Hill, Chapel Hill, NC, United States; ^4^ Department of Bioinformatics & Biostatistics, University of Louisville, Louisville, KY, United States; ^5^ Kentucky IDeA Networks of Biomedical Research Excellence (KY INBRE), Bioinformatics Core, University of Louisville, Louisville, KY, United States

**Keywords:** early-onset colon cancer, macrophages, immunology, obesity, metabolism

## Abstract

**Introduction:**

Obesity is a strong risk factor for early-onset colon cancer (EOCC) and is associated with chronic inflammation largely mediated by macrophages. The macrophage-specific metabolite itaconate promotes growth in several types of cancer; however, its role in colon cancer (CC) is unknown. Here, we investigate a tumor promoting link between obesity-related hormones and itaconate within the NOTCH4-GATA4-IRG1 pathway in EOCC.

**Methods:**

Patient tissue (n=20) was obtained and qRT-PCR, ELISA, and mass spectrometry were performed to evaluate *IRG1* expression (Human Immune-Responsive Gene 1, encoding ACOD1), ACOD1 expression (Cis-aconitate decarboxylase 1, enzyme producing itaconate), and itaconate concentration in human CC versus EOCC. RNA sequencing data from 5 sources in the USA and Europe were obtained to perform *IRG1*-related differential expression analysis (n=178), *IRG1*-related survival analysis (n=185), and differential expression analysis and survival analysis related to genes of the NOTCH4-GATA4-IRG1 pathway (n=371). Furthermore, tumor versus normal colon was compared and the interaction of tissue with sex, age, and body mass index (BMI) was investigated. A coculture model using two CC cell lines (HT-29 and SW480) and THP-1 cell line-derived M0 and M2-like macrophages was used to evaluate NOTCH4-GATA4-IRG1 pathway-related gene expression following treatment with obesity-related hormones (leptin, adiponectin) and itaconate derivatives.

**Results:**

Both ACOD1 and *IRG1* expression were elevated in human CC tissue compared to adjacent normal colon tissue. Normal colon itaconate levels were higher in EOCC patients compared to that in older patients. Plasma itaconate levels in CC patients correlated with their BMI. Survival was decreased in *IRG1*-positive stage IV CC. *IRG1*-associated gene expression within the NOTCH4-GATA4-IRG1 pathway differed in CC versus normal colon tissue: *GATA4*, *DLL4*, *VEGFA*, and *MAPK15* upregulation was associated with EOCC, while *ABCG5* and *GATA5* were downregulated in CCs and associated with higher BMI. Adiponectin and leptin treatment of macrophages cocultured with CC cells increased *IRG1* expression.

**Discussion:**

Obesity-related hormones can increase itaconate production in M2-like macrophages. *IRG1* expression and the NOTCH4-GATA4-IRG1 pathway are associated with EOCC, BMI, and patient survival. As a macrophage metabolite affecting inflammation, itaconate may have a particular immunotherapeutic role in patients with EOCC.

## 1 Introduction

Early-onset colon cancer (EOCC) has become one of the three most common causes of cancer death among those less than 50 years old in the United States; the underlying mechanisms are poorly understood (1). Simultaneously, obesity and metabolic dysfunction prevalence continue to increase among people in the United States, with more than 42% of adults over the age of 20 years being obese (2). The Nurses’ Health Study II demonstrated a direct link between obesity and EOCC risk among young women (3).

Chronic inflammation and metabolic overload in obese individuals lead to altered cellular metabolism and increased oxidative stress, both associated with colorectal carcinogenesis (4–7). The anti-inflammatory mitochondrial metabolite itaconate, a specific product of the Krebs cycle in macrophages, is a regulator of these mechanisms (8). Itaconate is a dicarboxylic acid and an enzymatic product of cis-aconitate decarboxylase 1 (ACOD1). It mediates carcinogenic effects in a variety of cancers, including glioma, melanoma, and ovarian carcinoma (9–11). Its production is associated with proteins regulating carcinogenesis as part of the NOTCH4-GATA4-IRG1 pathway (12, 13) (gene network shown in the Results section).. Itaconate’s role in human CC is not known (14).

In order to proliferate rapidly, cancer cells must reprogram their metabolism, which is a hallmark of cancer. There are several subtypes of CC characterized by different metabolic gene expression profiles. The CC subtype exhibiting downregulated lipid metabolism is linked to shorter survival duration (15, 16). The amount of fatty acid oxidation as an energy source also characterizes the phenotypes of tumor-associated macrophages (TAMs). These in turn, regulate oxidative stress and inflammation within the tumor microenvironment (17). Macrophage metabolism and therefore polarization determine either a more M1-like, pro-inflammatory or an M2-like anti-inflammatory phenotype (18). M1-like and M2-like macrophages both use mitochondrial oxidative and lipid metabolism to a variable extent. Itaconate is a metabolite normally found in proinflammatory M1-like macrophages; however, it has been measured in the M2-like subtype as well, as part of M2-like cell activation via glutamine metabolism (8, 19). In obese patients, metabolic dysfunction results in a chronic proinflammatory state which may cause metabolic reprogramming in tissue-resident macrophages, accelerating tumor growth and cancer onset (14).

In the CC tumor microenvironment, M2-like macrophages promote cancer progression by providing an anti-inflammatory environment. Advanced CC tumor stage and poor patient survival are associated with an increased M2/M1-like TAM ratio (20, 21). It is poorly understood as to whether itaconate produced by TAM contributes to this anti-inflammatory tumor microenvironment, CC onset, or progression. The aim of this study was to identify the role of obesity-related hormones and the macrophage-specific metabolite itaconate on CC metabolism within the NOTCH4-GATA4-IRG1 pathway in patients with sporadic colon adenocarcinoma and specifically in patients with EOCC. Therefore, we used a 4-pronged approach, **1)** utilizing human colon cancer tissues and plasma samples to determine itaconate levels, as well as protein expression of ACOD1, (itaconate producing enzyme) and gene expression of Human Immune-Responsive Gene 1 (*IRG1*, the gene encoding ACOD1), **2)** using the TCGA database to determine the effect of *IRG1* gene expression on survival, **3)** NOTCH4-GATA4-IRG1-pathway focused differential gene expression analysis, survival and tissue interaction analyses using several CC RNAseq datasets as outlined in the methods, and **4)** using a macrophage/CC cell line model to evaluate the effect of adiponectin, leptin and itaconate treatment on gene expression.

## 2 Materials and methods

Consent was obtained from human participants in accordance with the ethical standards of the institutional research committee and with the 1964 Helsinki declaration. Ethical approval for this study was obtained from the Human Subjects Research and Institutional Review Board (IRB) of the University of Louisville, (IRB# 97.0361).

### 2.1 Cell lines and reagents

The human CC cell lines HT-29 (RRID: CVCL_0320) and SW480 (RRID: CVCL_0546) and the human monocyte cell line THP-1 (RRID: CVCL_0006) were obtained from the American Type Culture Collection (ATCC). Authentication by short tandem repeat (STR) analysis was performed for all cell lines. THP-1 cells were used to create either M0 or anti-inflammatory M2-like macrophages in a 14-day cell culture model as previously described (22) and cocultured with either HT-29 or SW480 CC cells, respectively. RPMI-1640 medium (ATCC) with 10% fetal bovine serum (FBS) was used, and 1% L-glutamine as well as 1% antibiotics (penicillin-streptomycin) (ATCC) were added. Cells were kept at 37°C and 5% CO_2_. THP-1 monocytic cells were seeded in 24-well plate inserts at a concentration of 0.2x10^6^ and either HT-29 or SW480 CC cells were seeded into the wells at 0.2x10^6^ cells per well (Falcon 24-well plates, VWR).

Leptin (BioVendor R&D), adiponectin (BioVendor R&D), 4-octyl-itaconate (OI) (Sigma-Aldrich) and dimethyl itaconate (DI) (Sigma-Aldrich) were processed as manufacturer’s instructions advised. Final treatment concentrations were reached by adding phosphate buffered saline (PBS) if needed to receive 300 ng/ml leptin, 20 μg/ml adiponectin, 50 μg/ml 4OI and 50 μg/ml DI. Itaconate is a highly polar molecule that has to be transferred intracellularly to exert its anti-inflammatory effects. OI and DI are exogenous esterification derivatives of itaconate that are able to cross the cell membrane and are commercially available to study itaconate’s anti-inflammatory effects.

Cocultured cells were treated in a time-response experiment in technical duplicates. Cells were treated with either leptin (n=10), adiponectin (n=10), or itaconate derivatives 4OI (n=10), or DI (n=10). Negative controls of each treatment and time point were treated with 10 μl/ml PBS. Samples were harvested after 6 hours and 18 hours of cell treatment.

### 2.2 Patient tissue samples

Twenty consecutive patients (age range 40–81 years) were included undergoing curative initial resection. Inclusion criteria were a diagnosis of sporadic colon adenocarcinoma with histologic confirmation and a minimum BMI of 18.5 kg/m^2^. Patients were excluded in case of a diagnosis of Lynch-syndrome, familial adenomatous polyposis (FAP), inflammatory bowel disease, or prior neoadjuvant chemotherapy and/or radiation. Patient demographics are shown in **Supplementary Table 1**. For purposes of this study, “young” or EOCC will be defined as CC in an individual ≤ 50 years old.

Both adjacent normal colon tissue (>5cm distance from the CC) and CC tissue were collected from each patient. In addition, 10 ml peripheral blood were collected immediately pre-operatively. Samples were processed directly after harvest and stored at -80°C.

Four paired tissue samples of adjacent normal colon tissue and CC (N=8) were obtained from the University of North Carolina at Chapel Hill, three pairs from patients with EOCC (diagnosis below the age of 50 years) and one paired sample from a patient above the age of 50 years at diagnosis. Samples were processed as above and stored at -80°C.

### 2.3 Gene expression analysis

The RNeasy kit (Qiagen) was used to obtain total RNA from CC and normal colon tissue. After RNA quantification using spectrophotometry (NanoDrop 1000, Thermo Scientific), reverse transcription to cDNA was performed. Quantitative real-time PCR (qRT-PCR) was performed using a StepOnePlus Real-Time PCR System (Applied Biosystems). TaqMan Expression Assays (Applied Biosystems) were used with *18S* as the housekeeping gene (*ABCG8*: Hs00223690, *IRG1*: Hs00985781_m1, CD80:Hs01045161_m1, *CCL22*: Hs01574247_m1, *CXCL10*: Hs00171042_m1, *GATA5*: Hs00388359_m1, *IL10*: Hs00961622_m1, *IL1B*: Hs01555410_m1, IL6: Hs00174131_m1, *IL8*: Hs00174103_m1, *LCT*: Hs00158722_m1, *CD206*: Hs00267207_m1, *NFKB*: Hs00765730_m1, *PLIN4*: Hs00287411_m1, *PPARA*: Hs00947538_m1, *PPARG*: Hs01115513_m1, SERPINE: Hs00167155_m1, *TNF*: Hs00174128_m1, RNA*18S5*: Hs03928990_g1). For comparison of gene expression between samples ΔCT values were calculated. Results in macrophages were considered significant if demonstrated in combination with both CC cell lines. Results in CC cells were considered significant if shown with either M0 or M2-like macrophages.

### 2.4 Protein expression analysis using ELISA

The enzyme ACOD1 (encoding gene *IRG1*) was detected in normal colon tissue samples and CC by ELISA (IRG1-ELISA Kit, MyBioSource, Inc.). Protein concentrations are listed per µg total protein per respective sample.

### 2.5 Determination of itaconate levels using LC-MS/MS

Liquid chromatography with tandem mass spectrometry (LC-MS/MS) was used to determine itaconate levels in plasma, normal colon tissue and CC samples. An AB SCIEX API 4000TM tandem mass spectrometer (Concord) was coupled to Waters Acquity UPLC^®^ BEH C18 columns (2.1×100 mm, 1.7 μm), with VanGuard T3 precolumns (2.1×5 mm, 1.7 μm) (Waters). Buffer A (mobile phase) consisted of 0.1% formic acid-water and buffer B acetonitrile with the following mass spectrometry conditions: 500°C, ion spray voltage (IS): -4500V, curtain gas (CUR): 25psi, gas 1 (GS1): 50psi, gas 2 (GS2): 60psi.

### 2.6 Determination of itaconate levels using LC-MS/MS

RNA-seq data on human colon samples was acquired from The Cancer Genome Atlas (TCGA)(RRID: SCR_003193) (n=40) and the European Genome-Phenome Archive (EGA) (RRID: SCR_004944) (n=69) (23, 24). (EGA data *is hosted by the European Bioinformatics Institute (EBI) and the Centre for Genomic Regulation (CRG), under accession number EGAD00001000215.) Further data on human normal colon samples was obtained from the* Genotype-Tissue Expression (GTEx) Project database (GTEx Portal, dbGaP accession number phs000424.v8.p2) (RRID: SCR_013042). Additional RNA-seq data was collected from sequenced patient tissue samples from the University of Louisville (N=40) and the University of North Carolina at Chapel Hill (UNC) (N=7).

RNA-seq data on 371 sporadic CC samples and matched normal colon tissue samples for age, sex, and BMI was analyzed for 18,884 genes.

### 2.7 Differential expression and tissue interaction analyses

Specific *IRG1*-related differential gene expression (N=109), *IRG1*-related survival analysis (N=185) and NOTCH4-GATA4-IRG1-focused differential gene expression and survival analysis (N=362) were performed.

Data on forty CC samples with matching normal colon samples were obtained from The Cancer Genome Atlas (TCGA), each with raw gene counts for 60,483 gene locations (**Table 1**) (23). The European Genome-phenome Archive (EGA) supplied 69 additional CC cases with matching normal colon tissue as paired-end raw sequencing files (fastq) (24, 25). Each EGA sample data file contained an average of 33 million reads. FastQC (v.0.10.1) (RRID: SCR_014583) was used to determine read quality. Based on this, no sequence trimming had to be performed (26). Star (v.2.6) (RRID: SCR_005622) was used to align sequences to the human reference genome assembly (hg38) using an average 97% alignment rate across samples (27). HTSeq (v.0.10.0) (RRID: SCR_005514) using Gencode (v22) (RRID: SCR_014966) annotations (identical with the ones used to derive TCGA sample gene counts) was used to obtain read counts for gene regions (28, 29). Raw counts were produced for the same 60,483 gene locations. The files obtained from EGA and TCGA were merged into one data set and genes with low expression were filtered, resulting in 46,634 gene locations. The relative log expression (RLE) method was used to normalize combined raw read counts which were then input to a principal component analysis (PCA). This visually demonstrated appropriate separation of normal colon tissue and CC samples (**Supplementary Figure 1**).

**Table 1:** Number of patient samples (No.) for RNA sequencing (RNA-seq) data & respective analyses (differential gene expression & survival analysis).

RNA-seq dataset	Specific *IRG1*-related differential expression	Specific *IRG1*-related survival analysis	Differential expression analysis and survival analysis for NOTCH4-GATA4-IRG1 pathway
	No. patient samples	No. patient samples	No. patient samples
TCGA	40 cancer	185 cancer	176 cancer
EGA	69 cancer 69 adjacent nml colon		
UofL			16 cancer 15 adjacent nml colon
UNC			4 cancer 3 adjacent nml colon
GTEX			157 matched nml colon
Total	178	185	371
Tissue types	109 cancer 69 adjacent nml colon	185 cancer	196 cancer 175 nml colon ➔156 matched samples (cancer/nml colon)

Nml, normal; No., number of patient samples; TCGA, The Cancer Genoma Atlas; EGA, The European Genome-Phenome Archive; UofL, University of Louisville; UNC, University of North Carolina Chapel Hill; GTEX, Genotype Tissue Expression Project Database.

DEA for CC relative to normal tissue was performed using DESeq2 (RRID: SCR_015687) with its negative binomial regression model (30). The patient number was defined as a regression model covariate to account for sample pairing.

For tissue interaction analysis (N=362), 18,884 protein coding genes were tested. The parameters cancer versus normal, location (colon (general, right, left)), sex (male/female), age (younger, ≤50 years versus older, >50 years), and BMI (normal/overweight/obese) were included in the statistical model. Tumor versus normal colon was compared and the interaction of tissue with sex, age, and BMI was investigated.

### 2.8 Survival analysis

One hundred eighty-five TCGA colon tumor samples with information about patient survival were included for *IRG1*-related survival analysis (**Table 1**). Low expressed genes were filtered, and the RLE method used to normalize raw read counts. Significance levels and Kaplan-Meier curves were generated using R [R Foundation for Statistical Computing, survival package (v3.2.7)] to analyze the association of *IRG1* expression with patient survival (31, 32).

For survival analysis related to expression of inflammatory genes of the NOTCH4-GATA4-IRG1 pathway, we investigated 156 paired tissue samples (N=312) with CC and matching normal colon samples.

### 2.9 Statistics

Prism 9 (GraphPad Software, Inc.) software was used for statistical analysis. Results are reported as mean values ± standard deviation or 95% confidence intervals (CI).

Binominal data of gene expression in patient samples was analyzed using a McNemar test. Paired samples were compared with the Wilcoxon matched pairs test while the unpaired samples were compared using the Wilcoxon rank sum test. A 2-tailed p-value was calculated, level of significance 5%.

Spearman correlation analysis was also performed. Microsoft Excel Version 2017 was used to create graphs.

R was utilized for differential gene expression and survival analyses (32). Survminer 0.2.4. (RRID: SCR_021094) was used to determine cutoff points for significant gene expression (*IRG1*-positive samples) versus no gene expression (*IRG1*-negative samples).

## 3 Results

### 3.1 *IRG1* expression, ACOD1 expression and Itaconate levels in colon cancer patients

**Figure 1:**
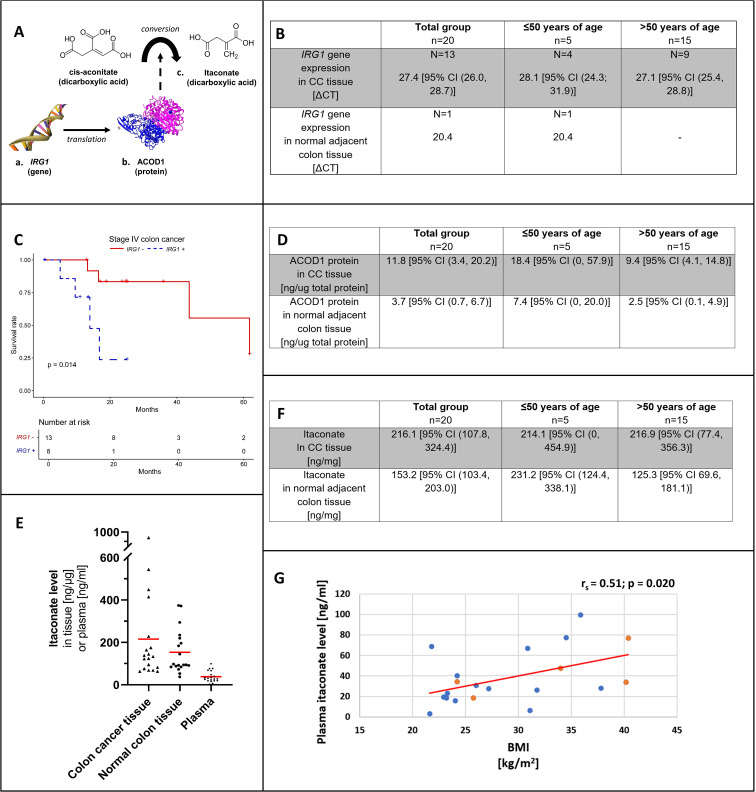
Overview on itaconate from gene to protein and anti-inflammatory metabolite **(A)**. *IRG1* gene expression **(B)** by patient age group in colon cancer tissue and adjacent normal colon. **(C)** shows the Kaplan-Meier curve for stage IV colon adenocarcinoma and colon cancer tissue *IRG1* gene expression (blue, dotted line) versus no *IRG1* gene expression (red, solid line). ACOD1 protein expression **(D)** by patient age group in colon cancer tissue and adjacent normal colon. **(E)** shows the distribution of itaconate levels with mean values (red) among patient tissue (colon cancer and adjacent normal colon) and corresponding patient plasma samples. Itaconate tissue levels **(F)** by patient age group in colon cancer tissue and adjacent normal colon. Correlation between plasma itaconate level and body mass index (BMI) among patients **(G)**. Levels of patients ≤50 years of age are represented in orange and those of patients >50 years of age are shown in blue. *CC, Colon cancer; IRG1, Immune-Responsive Gene 1; ACOD1, cis-aconitate decarboxylase 1*.

Itaconate metabolism was analyzed in patient colon tissue samples on three levels including (a) gene expression of *IRG1*, (b) protein expression of ACOD1 (enzyme responsible for itaconate production) and (c) by measuring the dicarboxylic acid itaconate and by investigating the role of *IRG1* gene expression in patient survival (**Figure 1A**).

### 3.2 *IRG1* gene expression and ACOD1 protein levels are increased in human colon cancer

In CC tissue, the mean ΔCT for *IRG1* gene expression was 27.4 [95% CI (26.0, 28.7)]. *IRG1* was expressed in 65% of CC samples and in only 5% of normal colon tissue samples with a ΔCT of 20.4 (chi-square: 10.083, p=0.002) (**Figure 1B**).

ACOD1 levels were higher in CC compared to normal colon (11.8 ng/ug [95% CI (3.4, 20.2 ng/ug)] versus 3.7 ng/ug [95% CI (0.7, 6.7 ng/ug)]; p=0.002), respectively. Mean ACOD1 concentration was higher in patients with EOCC compared to patients >50 years of age, with higher ACOD1 levels in both CC tissue and normal colon tissue; however, this did not reach statistical significance (**Figure 1D**).

### 3.3 Itaconate levels in normal colon tissue of young CC patients are higher than those seen in patients >50 years of age

**Figure 1E** is showing itaconate concentrations in paired patient samples (CC/normal colon tissue and plasma).

In both CC and normal colon samples, itaconate was detected showing high variability of concentrations in both tissue types. Overall CC tissue itaconate levels appeared higher; however, no significant difference was demonstrated (216.1 ng/mg [95% CI (107.8, 324.4)] versus 153.2 ng/mg [95% CI (103.4, 203.0)]; p=0.294) (**Figure 1F**). Normal colon itaconate levels were higher in individuals with EOCC compared to those >50 years old (231.2 ng/mg [95% CI (124.4, 338.1)] versus 125.3 ng/mg [95% CI 69.6, 181.1)]; p=0.026) (**Figure 1F**).

Plasma itaconate concentrations and BMI correlated positively (r_s_= 0.51; p=0.020) (**Figure 1G**). Itaconate plasma levels showed no correlation with neither CC tissue levels (r_s_= -0.20; p=0.414) nor normal colon tissue concentrations (r_s_= 0.19; p=0.433).

### 3.4 TCGA database analysis to determine the effect of *IRG1* expression on survival

#### 3.4.1 *IRG1* expression is linked to higher risk of death in stage IV colon cancer

The TCGA cohort (n=40) (**Table 1**) consisted of 48% men aged 40–90 years. In 39 out of 40 cases information on tumor stage was provided. Patients included showed stage I disease (N=5), stage II (N=22), stage III (N=5), and stage IV (N=7) disease. The EGA dataset did not provide metadata such as tumor stage or age at diagnosis.

Analyzing data of 109 individuals showed that *IRG1* expression was detected in 53% of CC samples and 25% of normal colon tissues (z = 4.68, p=<0.001). Upregulation of *IRG1* expression in CC samples relative to paired normal colon tissue was demonstrated (log_2_ FC=1.41, corrected p=0.03).

Across 185 CC tissue samples at different stages of tumor progression, detection of *IRG1* (*IRG1* expressed versus *IRG1* not expressed) had no effect on survival (X^2^ = 0, p=0.9). The number of events for each tumor stage are listed in **Supplementary Table 2**. At stage I no deaths occurred and survival was not affected at stage II or III (stage II X^2^ = 1.4, p=0.2; stage III X^2^ = 1.7, p = 0.2). The risk of death was increased for the subset of stage IV cancer patients showing a risk increase for detectable *IRG1* gene expression relative to no expression (X^2^ = 6.3, p=0.01). The Kaplan-Meier curve demonstrating stage IV survival is shown in **Figure 1C**.

### 3.5 NOTCH4-GATA4-IRG1 pathway – differential gene expression, survival and tissue interaction analyses

#### 3.5.1 Age- and BMI-related FABP6 and GATA5 expression in colon cancer is linked to poor overall survival

**Figure 2:**
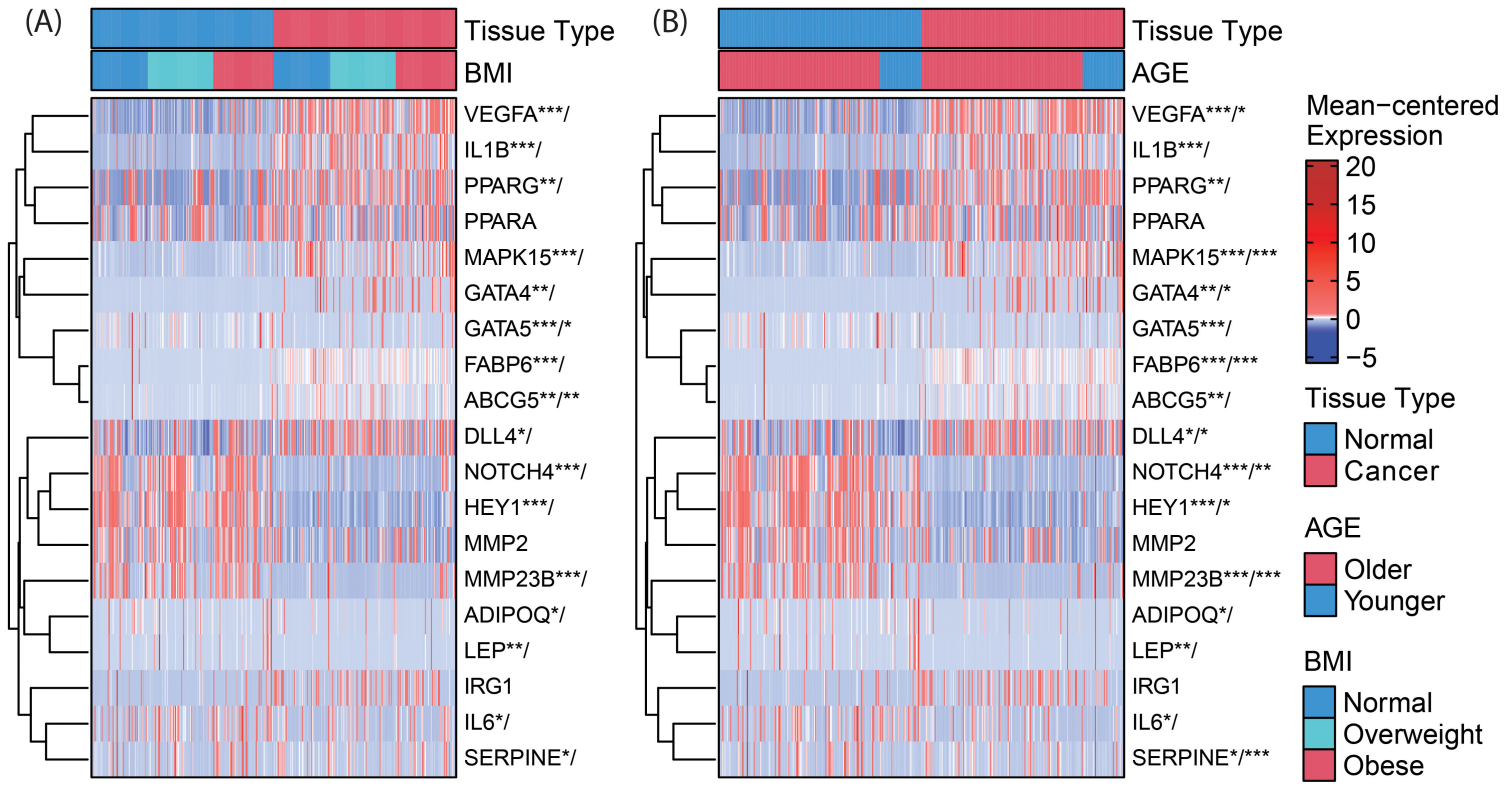
Clustered heatmaps (rows only) for differential gene expression and **(A)** BMI-by-tissue-type and **(B)** age-by-tissue-type interaction of genes within the NOTCH4-GATA4-IRG1 axis. Significance of annotated genes is shown as cancer tissue versus normal tissue/interaction for BMI (or age) with p-values * < *0.05*, ** < 0.01, and *** < 0.001. VEGFA, Vascular Endothelial Growth Factor A; IL1B, Interleukin 1 Beta; PPARG, Peroxisome Proliferator Activated Receptor Gamma; PPARA, Peroxisome Proliferator Activated Receptor Alpha; MAPK15, Mitogen-Activated Protein Kinase 15; GATA4, GATA Binding Protein 4; GATA5, GATA Binding Protein 5; FABP6, Fatty Acid Binding Protein 6; ABCG5, ATP Binding Cassette Subfamily G Member 5; DLL4, Delta Like Canonical Notch Ligand 4; NOTCH4, Notch Receptor 4; HEY1, Hes Related Family BHLH Transcription Factor With YRPW Motif 1; MMP2, Matrix Metallopeptidase 2; ADIPOQ, Adiponectin, C1Q And Collagen Domain Containing; LEP, Leptin; IRG1, Immunoresponsive Gene 1; IL6, Interleukin 6; SERPINE1, Serpin Family E Member 1.

**Figure 3:**
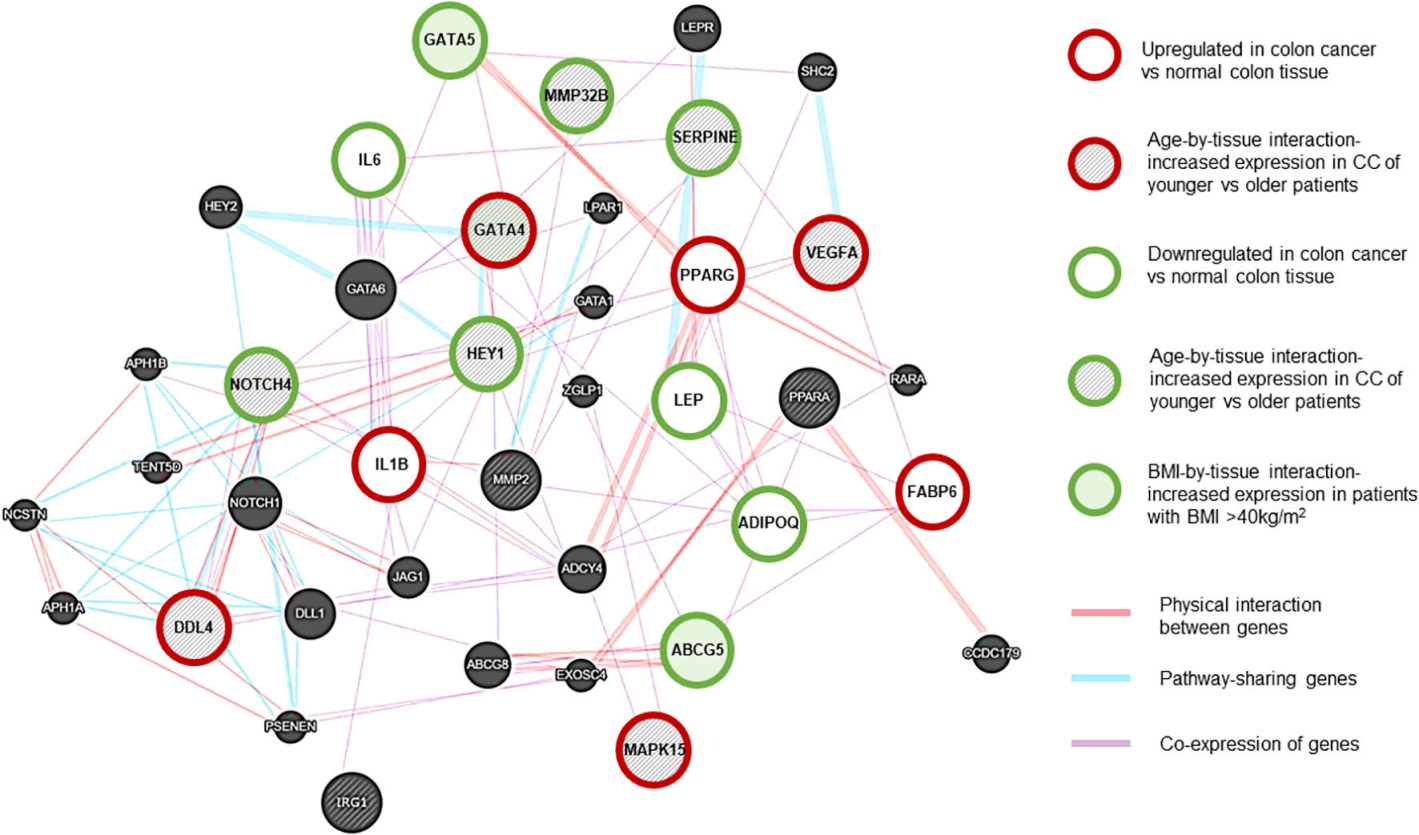
NOTCH4-GATA4-IRG1 network generated using Cytoscape (RRID: SCR_003032) (13). Gene associations are shown with different colors. *Purple lines: Coexpression of genes*. *Red lines: Physical interaction between genes*. *Blue lines: Pathway-sharing genes.* Red outline: genes with significant upregulation in CC vs normal tissue, Green outline: genes with significant downregulation in CC vs normal tissue. Grey-shaded stripes: age-by-tissue interaction. Green shaded: BMI by tissue interaction. (Based on paired samples, CC tissue N=181, normal colon tissue N=181.).

RNAseq data is presented as heat maps in **Figure 2** with corresponding values in **Supplementary Table 3**. The gene network involving NOTCH4-GATA4-IRG1 pathway genes is visualized in **Figure 3**.

In CC samples, the NOTCH4-GATA4-IRG1 pathway genes *DLL4*, *GATA4*, *IL1B*, *PPARG*, *VEGFA*, *MAPK15*, and *FABP6* (**Figure 3**) were upregulated compared to matched normal colon tissue of healthy individuals. The lowest mean overall expression was demonstrated for *IRG1*, therefore no significant difference was observed (**Supplementary Table 3**). The four target genes *DDL4*, *GATA4*, *VEGFA*, and *MAPK15* showed significant age-by-tissue interaction with increased expression in CC tissue of younger patients (**Figure 3**; **Supplementary Table 3**). Downregulation in CC tissue was demonstrated for *ABCG5*, *ADIPOQ*, *GATA5*, *HEY1*, *IL6*, *LEP*, *MMP23B*, *NOTCH4*, and *SERPINE*. A significant age-by-tissue interaction with upregulation in younger compared to older patients was shown for *HEY1*, *MMP23B*, *NOTCH4*, MAPK15 and *SERPINE*. Downregulation in younger versus older patients was demonstrated for *IL1B* and *PPARA*. BMI-by-tissue interaction with upregulation in patients with BMI >40 kg/m^2^ (N=60) was found for *ABCG5, GATA4*, and *GATA5*. In patients with obesity versus normal-weight patients downregulation was demonstrated for *NOTCH4* and *VEGFA* (**Figure 3**; **Supplementary Table 3**).

**Figure 4:**
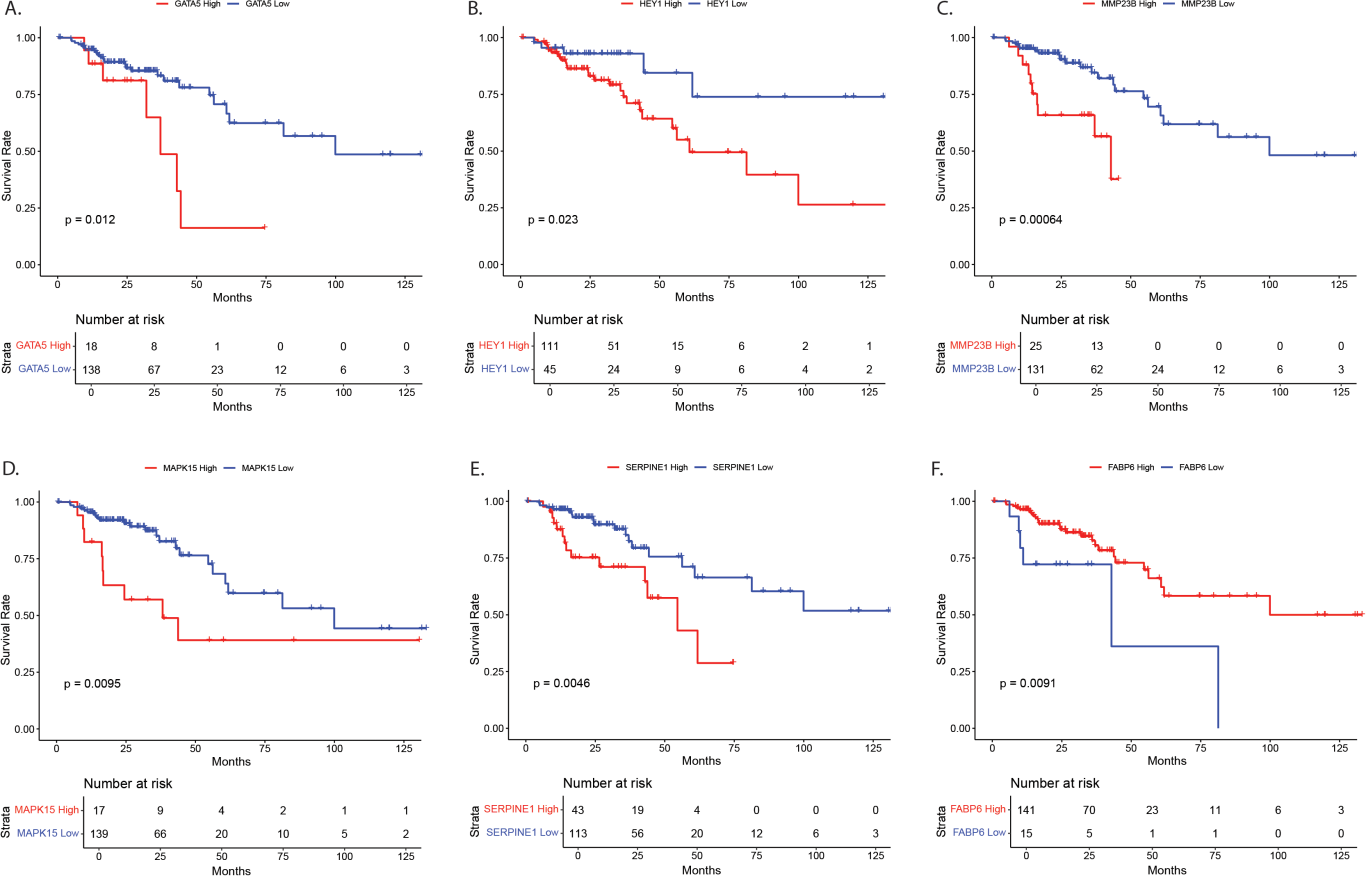
Survival analysis for NOTCH4-GATA4-IRG1 pathway target genes with significant age-by-tissue or BMI-by-tissue interaction for **(A)** *GATA5*, **(B)** *HEY1*, **(C)** *MMP23B*, **(D)** *MAPK15*, **(E)** *SERPINE1*, and **(F)** *FABP6*. Y-axes show survival probability, X-axes show time in months. GATA5, GATA Binding Protein 5; HEY1, Hes Related Family BHLH Transcription Factor With YRPW Motif 1; MMP23, Matrix Metallopeptidase 23; MAPK15, Mitogen-Activated Protein Kinase 15; SERPINE1, Serpin Family E Member 1; FABP6, Fatty Acid Binding Protein 6.

Genes with significant age-by-tissue or BMI-by-tissue interactions revealed that high *GATA5* (p=0.012), *HEY1* (p=0.023), *MMP23B (p=0.001)*, *MAPK15* (p=0.010) and *SERPINE1* (p=0.005) expression (**Figures 4A–E**) and low *FABP6* expression (p=0.010) (**Figure 4F**) were associated with decreased overall patient survival.

### 3.6 Macrophage/CC cell line coculture model to evaluate the effect of adiponectin, leptin and itaconate on gene expression

#### 3.6.1 Obesity-related hormones and itaconate induce procarcinogenic gene expression

**Figure 5:**
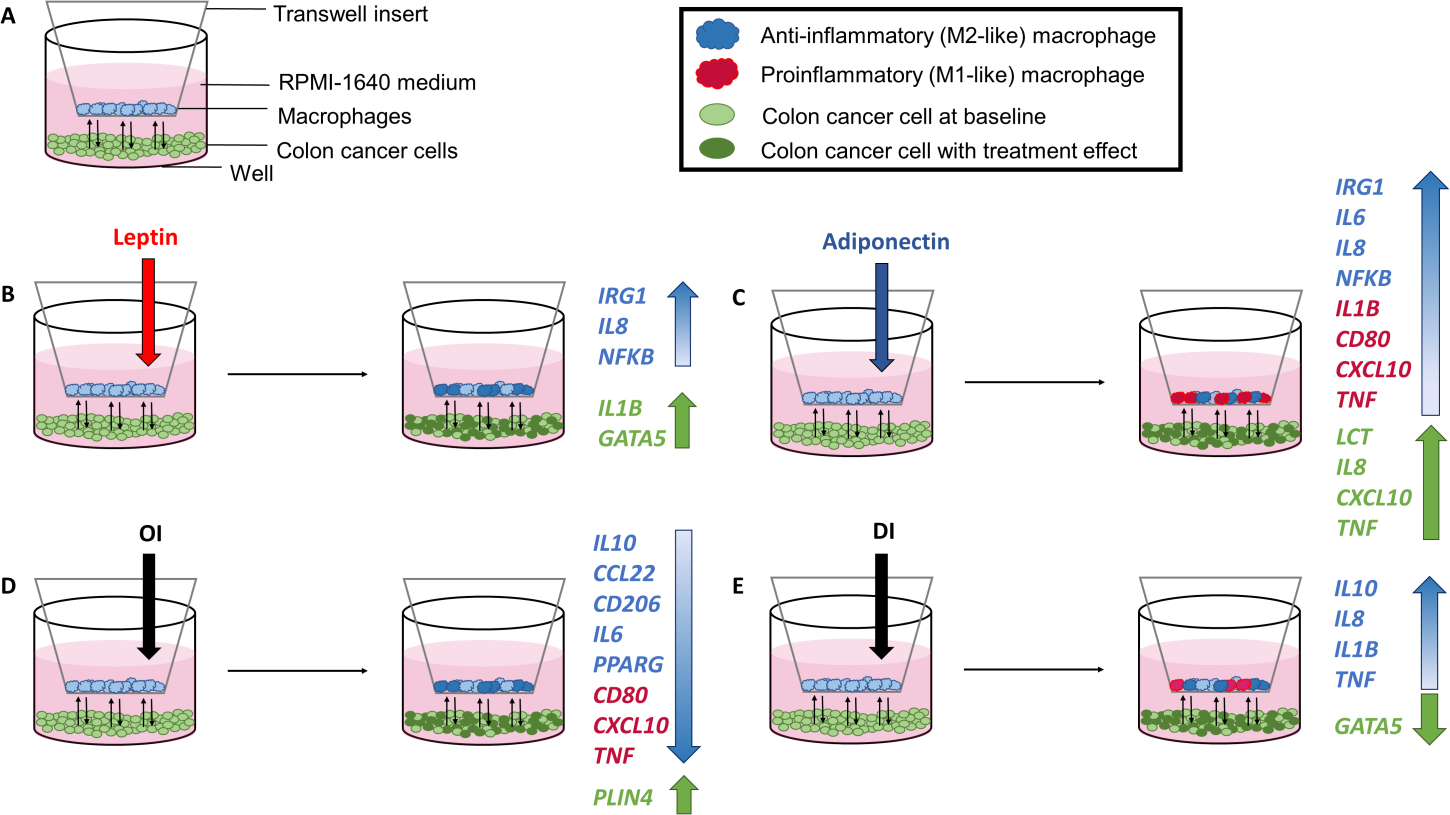
Coculture model with experimental setup **(A)** and cell treatments with corresponding gene expression results following leptin **(B)**, adiponectin **(C)**, OI (4-octyl itaconate) **(D)**, and DI (dimethyl itaconate) **(E)** treatment.

The coculture model with cell treatments and major gene expression changes is shown in **Figure 5**.

**Figure 6:**
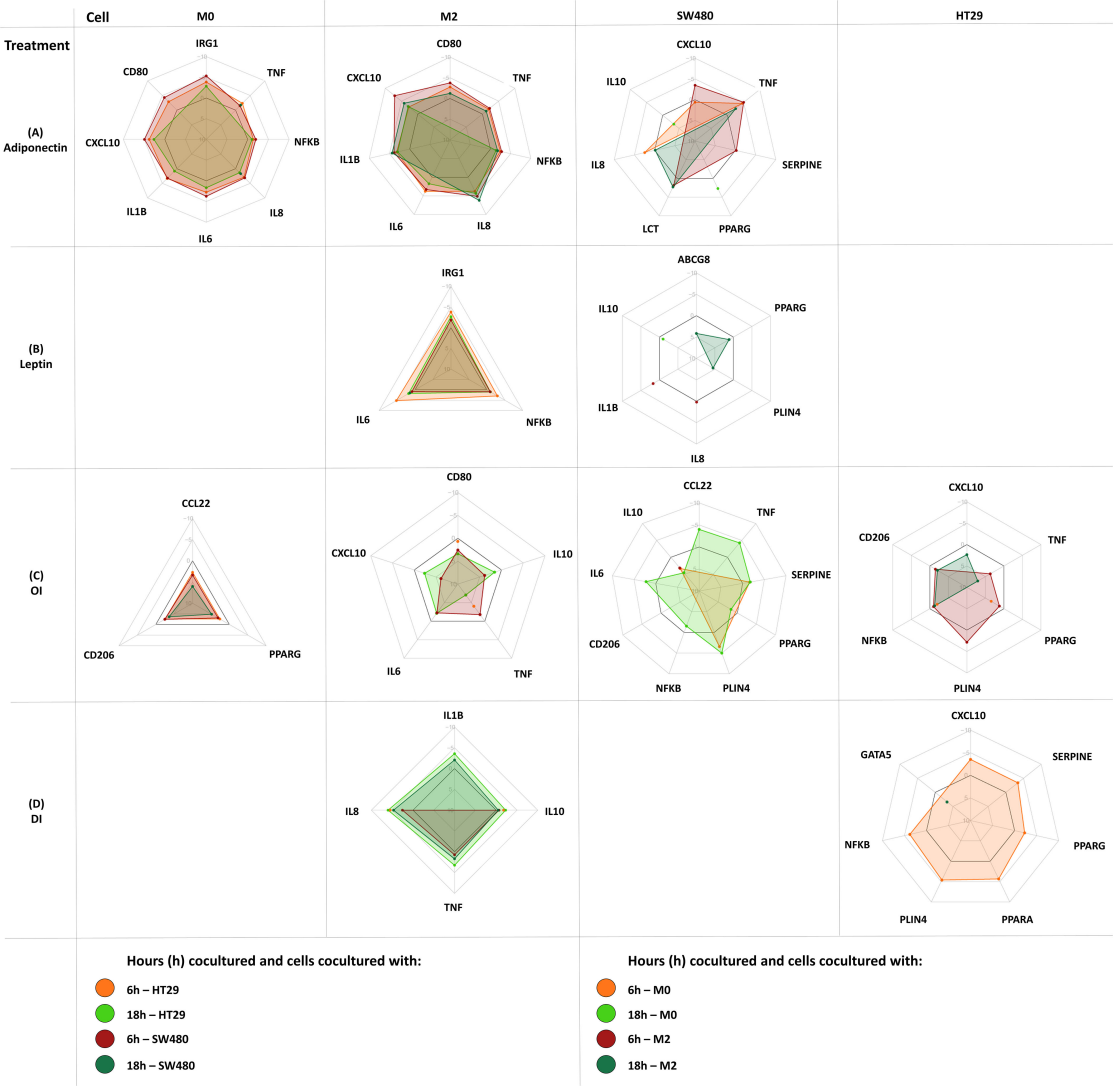
Gene expression changes in coculture following cell treatment (M0-macrophage, M2-macrophage as well as SW480, HT29 gene expression). Either M0 or M2 macrophages were cultured separately with either SW480 or HT29 CC cell lines and harvested after treatment at 6 hours and 18 hours. Cocultured cells were treated with either adiponectin **(A)**, leptin **(B)**, OI (4-octyl itaconate) **(C)**, or DI (dimethyl itaconate) **(D)**. Results are expressed as DDCT ± SEM. N=10 for each time point and treatment dose. The spider web line showing a DDCT of 0 is marked bold black (no change in gene expression). Upregulation is shown by a negative DDCT, while downregulations have positive DDCT values (DDCT values are shown on the vertical axis from -10 to +10). Single data points are shown as dots. Only statistically significant genes and time points are shown, for complete data see **Supplementary Tables 4**-**11**.

Corresponding radar plots are shown in **Figures 6**. All ΔCT values and FC are shown in **Supplementary Tables 4**-**11**.

#### 3.6.2 Leptin and adiponectin increase tumor promoting IRG1 and IL8 expression in macrophages

Gene expression following treatment with either adiponectin, leptin, 4OI or DI was assessed in macrophages. Significant changes were shown in both M0 and M2-like macrophages for all treatments except for leptin, which only affected M2 macrophages.

Adiponectin increased tumor promoting *IRG1* expression in M0 macrophages, as well as *IL6*, *IL8*, and *NFKB* expression in M0 and M2-like macrophages (**Figure 6A**). It upregulated proinflammatory gene expression including *CD80*, *CXCL10*, and *TNFA* in M0 and in M2-like macrophages. Procarcinogenic *IL1B* was upregulated by adiponectin in M2 macrophages.

Leptin only increased expression of *IRG1*, *IL8*, and *NFKB* in M2-like macrophages (**Figure 6B**).

#### 3.6.3 4OI and DI enhance anti-inflammatory gene expression in macrophages

In M0 macrophages, 4OI downregulated the monocyte and T-cell chemoattractant *CCL22* and anti-inflammatory marker *CD206*, and exerted tumor promoting effects by directly downregulating *PPARG* (**Figure 6C**). M2-like macrophages demonstrated a predominantly anti-inflammatory profile following 4OI treatment with downregulation of proinflammatory *CD80*, *CXCL10*, *TNFA*, and *IL6* (**Figure 6C**). While 4OI downregulated anti-inflammatory *IL10* expression in M2 macrophages, DI upregulated *IL10*. DI also exerted further anti-inflammatory effects by upregulating *IL8* in M0 and in M2 macrophages (**Figures 6C, D**).

#### 3.6.4 Leptin, adiponectin and 4OI alter PPARG expression and induce tumor promoting mediator expression in colon cancer cells

Adiponectin, leptin, 4OI, and DI affected gene expression in HT29 and SW480 CC cell line cells (**Figure 6**).

Adiponectin only affected gene expression in SW480 and upregulated *PPARG*, *CXCL10*, *IL8*, *LCT*, *SERPINE* and *TNFA*, and downregulated *IL10* (**Figure 6A**).

Only leptin treatment had an impact on *ABCG8* and *IL1B* expression. Leptin upregulated *GATA5* and downregulated *CCL22*, *IL10*, *IL8*, *PLIN4*, and *PPARG* (**Figure 6B**).

4OI exerted tumor promoting effects in both cancer cell lines by downregulating *NFKB* and *PPARG* in coculture with either M0 or M2 macrophages. *PLIN4* was upregulated in both CC cell lines following 4OI treatment (**Figure 6C**).

In SW480, 4OI showed overall proinflammatory effects. In HT29, anti-inflammatory responses were observed with downregulation of *CXCL10* and *TNFA* (**Figure 6C**).

DI upregulated *CXCL10*, *NFKB*, *PLIN4*, PPARA, *PPARG*, and *SERPINE*, and downregulated *GATA5* in HT29, and *IL8* and *NFKB* in SW480 (**Figure 6D**).

## 4 Discussion

Itaconate is a macrophage-specific metabolite and part of the NOTCH4-GATA4-IRG1 pathway, a subset of signaling pathways associated with cell differentiation and proliferation in CC. It reduces oxidative stress as an anti-inflammatory mediator (8, 17, 33). Itaconate’s tumor- promoting effects have been previously demonstrated in different types of cancers (9–11). It serves as an anti-inflammatory counterpart in the chronic proinflammatory state of obesity, which potentially defines it as a linking mediator of obesity, metabolic dysfunction, and cancer.

There are currently insufficient data available on *IRG1*-related expression and itaconate levels as part of the NOTCH4-GATA4-IRG1 pathway in CC. This study demonstrates that in individuals with obesity and sporadic CC, itaconate may affect cancer development. On the background of continuously increasing rates of obesity and EOCC, itaconate may play a key role in younger individuals with CC.

Gene expression of *IRG1*, which encodes the itaconate producing enzyme ACOD1, was more frequently detected in CC tissue samples (65%) versus matched normal adjacent colon tissue (5%). RNA-seq data results confirmed these findings (53% versus 25%). Also ACOD1 protein expression was significantly higher in CC versus normal colon tissue. Therefore, *IRG1* may be amplified in sporadic CC and may represent an oncogene target. Similar ACOD1 protein expression patterns have been found in cancers such as human glioma, correlating with recurrence-free survival and cancer stage (11). ACOD1 expression was classified as either low or high in this study, without reporting quantitative expression levels (11). In patients with ovarian carcinoma, monocytes harvested from ascites fluid showed enhanced *IRG1* expression, enforcing peritoneal tumor progression (9). In various murine cancer cell lines (melanoma, lung carcinoma, colon adenocarcinoma), *IRG1* mRNA was detected (9). These findings suggest that tumor promoting effects through *IRG1*-related mechanisms may not be CC specific. The important role of macrophage metabolism and itaconate in CC; however, underlines the importance of immunosuppression via itaconate within the NOTCH4-GATA4-IRG1 pathway in CC (34, 35). Oxidative phosphorylation in TAMs is directly impacted by itaconate. This is accompanied by reactive oxygen species (ROS) production and macrophage polarization (9). ROS mediate cell proliferation, migration, and angiogenesis through several transcription factors and processes of autophosphorylation, including NF-κB, vascular endothelial growth factor receptor 2 (VEGFR2), and mitogen-activated protein kinases (MAPKs) (36). Development of colon polyps with malignant potential and poor patient survival in CC are associated with M2-like TAMs (37, 38). The relationship of NOTCH4-GATA4-IRG1 signaling, itaconate metabolism, and an anti-inflammatory macrophage phenotype in CC has yet to be investigated further.

EOCC, metabolic dysfunction, and obesity may be linked by itaconate metabolism. This study provides evidence for a link between obesity and systemic itaconate levels by demonstrating a correlation of BMI and preoperative plasma itaconate concentrations (r_s_=0.51; p=0.020). In individuals with elevated BMI and CC, the chronic proinflammatory state may be linked to an itaconate-producing macrophage phenotype derived from peripheral monocytes. In individuals with obesity, a phenotypic switch of adipose tissue-infiltrating macrophages is reported, which enhances inflammation, provokes insulin resistance and the onset of diabetes (39, 40). This switch mechanism could affect systemic itaconate concentrations and therefore CC risk as well.

A limitation of the correlation analysis shown in this study is that causality between itaconate and BMI cannot be confirmed and the sample size does not allow for concluding an empirical correlation. The observed correlations are primarily associative and future mechanistic studies with controlled experiments are necessary to establish causality. Finding a correlation that is significant despite these limitations, however, suggests that in patients with higher BMI and CC systemic itaconate may be a target of interest.

Comparing EOCC tissue versus that of patients with sporadic CC >50 years old revealed that a higher itaconate concentration was demonstrated in normal colon tissue of younger individuals with EOCC. There was no difference in CC itaconate concentrations between younger and older (>50 years of age) patients. These findings have not been previously reported. Due to the limited sample size, however, conclusions are limited, and findings cannot necessarily be generalized across the large and diverse population of colon cancer patients. As evidence suggests that colon cancer onset and progression are multifactorial, our results may only be significant for a certain subset of individuals with colon cancer. Despite the highly variable itaconate concentrations measured among colon samples, however, a significant difference was demonstrated. Therefore, additionally to itaconate’s basic role in CC, a particular effect on the early onset of CC may be related to itaconate, which should be further investigated.

Microsatellite-instability (MSI) affects tumor metabolism and clinical outcome in patients with CC. The MSI status correlates with the tumor location within the colon (41–43). Furthermore, MSI tumors show abnormal protein translation, which is triggering immune responses (44). Lacking metadata including MSI status marks another limitation of this study. In addition, small sample size did not allow for correlation of ACOD1 and itaconate levels with tumor stage.

Higher *IRG1* expression in CC versus normal colon samples as well as higher levels of itaconate itself in CC tissue was demonstrated by differential gene expression analysis. This suggests potential effects of *IRG1* expression in cancer growth and development. Only in patients with stage 4 CC, an effect of *IRG1* cancer tissue expression on patient survival was found. In earlier stages of CC, the limited number of patients and a varying sensitivity of the respective sequencing technique, which is negatively affecting the detection of the generally low expression of IRG1, may mask a potential effect. Standardized sequencing methods and tissue quality suggest that data sets are feasibly comparable; however, expression differences between samples may be masked due to variations in gene expression resolution. This can negatively affect the validity of differential expression calls for genes that show low expression in general, especially when a number of highly expressed outliers dominate a sample. This bias can mask expression of low expression genes and no significant difference in differential expression can be found. Our largest set of genomic data (N=380) did not confirm initial *IRG1* expression results. *IRG1*-associated gene expression of the NOTCH4-GATA4-IRG1 pathway; however, was altered significantly. *GATA4*, *DLL4*, *VEGFA*, and *MAPK15* were all upregulated in CC and seem to be associated with a younger patient age. *ABCG5* and *GATA5* were downregulated in CC and shown to be associated with higher BMI. A limitation of this analysis is the fact that CC and part of the normal colon samples were matched and were not obtained from the same patient, which can potentially mask differences in gene expression between tissues. To minimize this bias, however, we used adjacent normal colon tissue data where available, and used GTEx as a resource for normal colon samples matched for sex, age, and BMI.

The role of obesity-related hormones can have a significant impact on cell metabolism and therefore macrophage polarization, but their role in EOCC is mostly unknown. Our results suggest that adiponectin induces a mostly proinflammatory response in M0 macrophages with a simultaneous increase in *IRG1* expression, which may have a significant effect in early cancer development. Leptin upregulated *IRG1* in already polarized M2-like macrophages, and therefore exerted potential effects during later tumor stages. As a known key mechanism in CC development, OI mediated tumor promoting effects by downregulating *PPARG* in M0 macrophages and by enhancing the anti-inflammatory profile in M2-like macrophages (45). DI showed a mixed response by upregulating *IL8* and *IL1B* as a potential mechanism in CC (46, 47).

Itaconate and its related procarcinogenic mechanisms within the NOTCH4-GATA4-IRG1 pathway could represent a novel immunomodulatory target in TAM to break the NOTCH4 signaling cascade that enhances anti-inflammatory macrophage polarization. This could combat tumor-induced immunosuppression and tumor progression in individuals with obesity and CC and in patients with EOCC. Furthermore, suppression of IRG1 and obesity hormone-associated gene expression in macrophages could perhaps induce cancer remission or inhibit tumor growth, as well as prolong survival in advanced stage cancers. Our data suggest that TAM metabolism and its production of itaconate in EOCC may promote cancer progression.

*IRG1* is amplified in sporadic CC. ACOD1 protein levels and itaconate concentrations in colon tissue differ between age groups. These findings provide new insight into EOCC-related TAM metabolism. In patients with elevated BMI and CC, itaconate may have a immunotherapeutic role. Studies investigating a larger number of subjects are necessary to determine the role of itaconate in metabolic dysfunction and EOCC. Itaconate and the NOTCH4-GATA4-IRG1 pathway may represent potential immunotargets in EOCC.

## Data Availability

The datasets (TCGA, EGA, GTEx) presented in this study can be found in online repositories. The names of the repository/repositories and accession number(s) can be found in the article/**Supplementary Material**. Additional datasets included in this study are available upon request.
