# Supplementary material for: Itaconate and obesity-related hormones promote tumor progression – new insights on metabolic dysfunction in early-onset colon cancer
Source: Front Immunol. 2025 Jun 9;16:1572985. doi: 10.3389/fimmu.2025.1572985 (PMC12183228; doi:10.3389/fimmu.2025.1572985)
Supplement: Supplementary file 1 [file DataSheet1.docx]

Supplementary Material

# Supplementary Figures and Tables

## Supplementary Figures


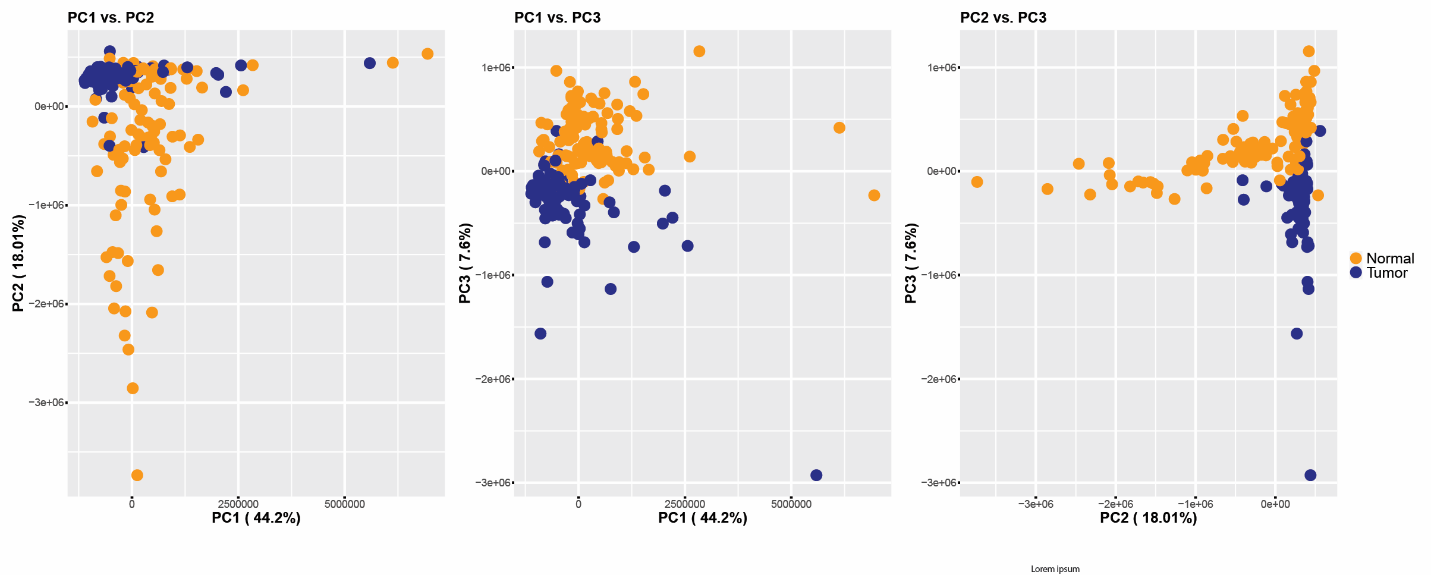


**Supplementary Figure 1.** Principal component analysis on tumor and normal tissue samples for specific IRG1-related differential expression. A clear separation between normal colon data (“Normal”, orange) and colon cancer data (“Tumor”, blue) could be demonstrated.

## Supplementary Tables

|  | **Total Group**  n=20 | **≤50 years of age**  n=5 | **>50 years of age**  n=15 |
| --- | --- | --- | --- |
| Gender [% men] | 55 | 40 | 60 |
| Mean age [years] | 65.9 ± 14.9 | 45.6 ± 4.2 | 72.6 ± 9.9 |
| Tumor stage^a^ [N] | - I (7) - II (9) - III (3) - IV (1) | - I (2) - II (3) | - I (5) - II (6) - III (3) - IV (1) |
| Mean body mass index (BMI)^b^ [kg/m^2^] | 29.0 ± 6.3 | 32.9 ± 7.7 | 27.8 ± 5.5 |
| Normal weight [%] | 40 | 20 | 46.7 |
| Overweight [%] | 15 | 20 | 13.3 |
| Obesity class 1 [%] | 25 | 20 | 26.7 |
| Obesity class 2 [%] | 10 | 0 | 13.3 |
| Obesity class 3 [%] | 10 | 40 | 0 |

**Supplementary Table 1.** Clinical characteristics of patients (n=20) providing paired colon cancer and normal colon samples for qRT-PCR, ELISA and mass spectrometry

*^a^American Joint Committee on Cancer (*AJCC*) staging*

*^b^**Body mass index [kg/m^2^] according to class of obesity (WHO classification) (22):*

*Normal weight: 18.5-24.9*

*Overweight: 25-30*

*Obesity class 1: 30-35*

*Obesity class 2: 35-40*

*Obesity class 3: >40*

| Stage | Deceased | Living |
| --- | --- | --- |
| I | 0 | 28 |
| II | 11 | 63 |
| III | 14 | 48 |
| IV | 8 | 13 |

**Supplementary Table 2.** Number of events at different tumor stages.

| **Gene** | **Mean  overall reads** | **Differential expression (Colon Cancer vs. Matched normal colon of healthy individuals) [log_2_ FC]** | **p-value** | **Age (≤50 years)-by-tissue interaction** | **p-value** | **BMI (>40 kg/m^2^)-by-tissue interaction** | **p-value** |
| --- | --- | --- | --- | --- | --- | --- | --- |
| ABCG5 | 38.1 | -1.3 | 0.004* | 0.1 | 0.775 | 2.1 | <0.001* |
| ADIPOQ | 404.5 | -1.8 | 0.038* | -1.7 | 0.086 | 0.5 | 0.606 |
| DLL4 | 1013.2 | 0.5 | 0.006* | 0.8 | <0.001* | -0.4 | 0.053 |
| GATA4 | 54.0 | 2.7 | <0.001* | 2.2 | 0.007* | 1.7 | 0.036* |
| GATA5 | 23.8 | -3.3 | <0.001* | 0.4 | 0.488 | 1.9 | 0.001* |
| HEY1 | 350.5 | -1.3 | <0.001* | 0.8 | <0.001* | -0.1 | 0.508 |
| IL1B | 492.1 | 1.7 | <0.001* | -1.7 | <0.001* | 0.1 | 0.873 |
| IL6 | 290.2 | -1.3 | 0.004* | 0.2 | 0.720 | -0.5 | 0.321 |
| IRG1 | 1.4 | -0.3 | 0.698 | -1.4 | 0.143 | 0.3 | 0.748 |
| LEP | 141.7 | -2.0 | 0.008* | 0.7 | 0.435 | 0.9 | 0.306 |
| MMP2 | 13751.3 | -0.8 | 0.002* | 0.4 | 0.224 | -0.2 | 0.444 |
| MMP23B | 30.1 | -2.5 | <0.001* | 2.1 | <0.001* | -0.2 | 0.547 |
| NOTCH4 | 1087.8 | -1.1 | <0.001* | 1.1 | <0.001* | -0.5 | 0.030* |
| PPARA | 2265.5 | 0.1 | 0.441 | -0.4 | 0.032* | -0.1 | 0.750 |
| PPARG | 2103.2 | 0.7 | 0.006* | -0.0 | 0.964 | 0.1 | 0.686 |
| SERPINE | 2954.9 | -1.2 | 0.003* | 2.2 | 0.001* | -0.3 | 0.529 |
| VEGFA | 6061.6 | 0.8 | <0.001* | 0.7 | 0.001* | -0.4 | 0.030* |
| MAPK15 | 248.9 | 1.9 | <0.001* | 1.8 | <0.001* | -0.2 | 0.674 |
| FABP6 | 270.4 | 5.0 | <0.001* | -3.5 | <0.001* | 0.3 | 0.601 |

**Supplementary Table 3.** Gene expression (normalized read counts), differential gene expression and age-by-tissue and BMI-by-tissue interaction of genes within the NOTCH4-GATA4-IRG1 axis.

*Significant results are shaded.*

*FC = fold change; BMI = body mass index*

|  | | | | | | | | | | | | | | | | | | | |
| --- | --- | --- | --- | --- | --- | --- | --- | --- | --- | --- | --- | --- | --- | --- | --- | --- | --- | --- | --- |
| Cell lines/  Treatment length | | **ΔCT** | **s.e.m** | **ΔΔCT** | **s.e.m** | **FC** | **p** | **ΔCT** | **s.e.m** | **ΔΔCT** | **s.e.m** | **FC** | **p** | **ΔCT** | **s.e.m** | **ΔΔCT** | **s.e.m** | **FC** | **p** |
|  |  | **IRG1** |  |  |  |  |  | **CD80** |  |  |  |  |  | **CXCL10** | |  |  |  |  |
| **M0/HT29** | L3 | 24.37 | 0.42 | -3.86 | 1.04 | 14.5 | 0.002 | 20 | 0.16 | -2.86 | 0.4 | 7.3 | <0.001 | 20.28 | 0.21 | -3.78 | 0.52 | 13.7 | <0.001 |
|  | L4 | 30.2 | 0.42 | -2.86 | 1.04 | 7.3 | 0.014 | 22.41 | 0.16 | -0.64 | 0.4 | 1.6 | 0.129 | 22.47 | 0.21 | -2.63 | 0.52 | 6.2 | <0.001 |
| *Overall p value* | |  |  |  |  |  | ***<0.001*** |  |  |  |  |  | ***<0.001*** |  |  |  |  |  | ***<0.001*** |
| **M0/SW480** | L3 | 21.24 | 0.31 | -5.34 | 0.77 | 40.6 | <0.001 | 16.31 | 0.29 | -4.3 | 0.72 | 19.8 | <0.001 | 18.65 | 0.5 | -4.91 | 1.22 | 30 | 0.002 |
|  | L4 | 24.52 | 0.31 | -0.16 | 0.77 | 1.1 | 0.835 | 22.77 | 0.33 | -0.54 | 0.74 | 1.5 | 0.478 | 25.19 | 0.65 | -1.27 | 1.29 | 2.4 | 0.343 |
| *Overall p value* | |  |  |  |  |  | ***<0.001*** |  |  |  |  |  | ***<0.001*** |  |  |  |  |  | ***0.005*** |
| **M2/HT29** | L3 | 25.36 | 0.5 | -2.01 | 1.22 | 4 | 0.121 | 18.45 | 0.3 | -2.77 | 0.74 | 6.8 | 0.002 | 19.09 | 0.45 | -2.74 | 1.1 | 6.7 | 0.024 |
|  | L4 | 26.02 | 0.56 | -2.14 | 1.24 | 4.4 | 0.107 | 19.66 | 0.3 | -1.14 | 0.74 | 2.2 | 0.144 | 20.69 | 0.45 | -2.95 | 1.1 | 7.8 | 0.016 |
| *Overall p value* | |  |  |  |  |  | *0.092* |  |  |  |  |  | ***0.004*** |  |  |  |  |  | ***0.007*** |
| **M2/SW480** | L3 | 23.86 | 0.64 | -8.21 | 1.58 | 296.4 | <0.001 | 18.44 | 0.19 | -3.76 | 0.47 | 13.5 | <0.001 | 16.64 | 0.28 | -7.11 | 0.68 | 138 | <0.001 |
|  | L4 | 30.16 | 0.64 | -2.99 | 1.58 | 7.9 | 0.076 | 21.14 | 0.19 | -1.23 | 0.47 | 2.3 | 0.019 | 18.79 | 0.28 | -4.18 | 0.68 | 18.1 | <0.001 |
| *Overall p value* | |  |  |  |  |  | *<0.001* |  |  |  |  |  | ***<0.001*** |  |  |  |  |  | ***<0.001*** |
|  |  | **IL8** |  |  |  |  |  | **NFkB** |  |  |  |  |  | **TNF** |  |  |  |  |  |
| **M0/HT29** | L3 | 11.86 | 0.16 | -2.9 | 0.4 | 7.5 | <0.001 | 15.02 | 0.19 | -1.37 | 0.47 | 2.6 | 0.01 | 18.03 | 0.18 | -2.33 | 0.44 | 5 | <0.001 |
|  | L4 | 14.97 | 0.16 | -1.37 | 0.4 | 2.6 | 0.003 | 15.68 | 0.19 | -1.04 | 0.47 | 2 | 0.043 | 20.29 | 0.2 | -0.65 | 0.45 | 1.6 | 0.174 |
| *Overall p value* | |  |  |  |  |  | **<0.001** |  |  |  |  |  | **0.008** |  |  |  |  |  | **<0.001** |
| **M0/SW480** | L3 | 10 | 0.24 | -3.14 | 0.6 | 8.8 | <0.001 | 14.02 | 0.25 | -1.95 | 0.61 | 3.9 | 0.006 | 17.84 | 0.19 | -1.5 | 0.47 | 2.8 | 0.007 |
|  | L4 | 11.96 | 0.27 | -1.6 | 0.61 | 3 | 0.019 | 16.28 | 0.25 | -0.54 | 0.61 | 1.5 | 0.388 | 17.04 | 0.22 | -1.19 | 0.48 | 2.3 | 0.027 |
| *Overall p value* | |  |  |  |  |  | **<0.001** |  |  |  |  |  | **0.001** |  |  |  |  |  | **0.005** |
| **M2/HT29** | L3 | 13.94 | 0.29 | -3.78 | 0.7 | 13.8 | <0.001 | 15.13 | 0.26 | -2.47 | 0.63 | 5.5 | 0.001 | 17.25 | 0.23 | -1.91 | 0.57 | 3.8 | 0.004 |
|  | L4 | 15.09 | 0.29 | -4.38 | 0.7 | 20.9 | <0.001 | 16.17 | 0.26 | -1.4 | 0.63 | 2.6 | 0.04 | 18.49 | 0.23 | -0.61 | 0.57 | 1.5 | 0.3 |
| *Overall p value* | |  |  |  |  |  | ***<0.001*** |  |  |  |  |  | ***0.015*** |  |  |  |  |  | ***0.01*** |
| **M2/SW480** | L3 | 13.74 | 0.17 | -5.14 | 0.41 | 35.1 | <0.001 | 15.03 | 0.16 | -2.78 | 0.39 | 6.9 | <0.001 | 15.73 | 0.19 | -2.22 | 0.47 | 4.7 | <0.001 |
|  | L4 | 14.54 | 0.19 | -6.22 | 0.42 | 74.5 | <0.001 | 16.23 | 0.18 | -1.79 | 0.4 | 3.5 | <0.001 | 18.22 | 0.19 | -1.15 | 0.47 | 2.2 | 0.026 |
| *Overall p value* | |  |  |  |  |  | ***<0.001*** |  |  |  |  |  | ***<0.001*** |  |  |  |  |  | ***<0.001*** |
|  |  | **PPARG** | |  |  |  |  | **IL10** |  |  |  |  |  | **CCL22** |  |  |  |  |  |
| **M0/HT29** | L3 | 16.42 | 0.13 | -0.1 | 0.33 | 1.1 | 0.76 | 21.86 | 0.17 | -0.87 | 0.41 | 1.8 | 0.048 | 18.43 | 0.12 | -0.49 | 0.29 | 1.4 | 0.105 |
|  | L4 | 17.7 | 0.13 | 0.59 | 0.33 | -1.5 | 0.088 | 22.01 | 0.17 | -0.16 | 0.41 | 1.1 | 0.701 | 18.4 | 0.12 | -0.01 | 0.29 | 1 | 0.964 |
| *Overall p value* | |  |  |  |  |  | *0.215* |  |  |  |  |  | *0.126* |  |  |  |  |  | *0.258* |
| **M0/SW480** | L3 | 17.01 | 0.28 | 0.86 | 0.69 | -1.8 | 0.23 | 21 | 0.38 | -0.09 | 0.93 | 1.1 | 0.928 | 16.48 | 0.24 | -1.32 | 0.59 | 2.5 | 0.04 |
|  | L4 | 18.7 | 0.28 | 0.44 | 0.69 | -1.4 | 0.53 | 24.28 | 0.38 | 0.82 | 0.93 | -1.8 | 0.39 | 20.59 | 0.24 | 0.7 | 0.59 | -1.6 | 0.251 |
| *Overall p value* | |  |  |  |  |  | *0.395* |  |  |  |  |  | *0.681* |  |  |  |  |  | *0.067* |
| **M2/HT29** | L3 | 19.13 | 0.18 | 1.28 | 0.43 | -2.4 | 0.009 | 17.45 | 0.32 | -1.11 | 0.79 | 2.2 | 0.18 | 18.96 | 0.19 | -1.31 | 0.47 | 2.5 | 0.013 |
|  | L4 | 18.86 | 0.18 | -0.34 | 0.43 | 1.3 | 0.44 | 17.88 | 0.32 | -0.27 | 0.79 | 1.2 | 0.738 | 19 | 0.19 | -0.27 | 0.47 | 1.2 | 0.565 |
| *Overall p value* | |  |  |  |  |  | *0.025* |  |  |  |  |  | *0.377* |  |  |  |  |  | *0.036* |
| **M2/SW480** | L3 | 18.67 | 0.13 | -0.54 | 0.31 | 1.4 | 0.103 | 19.55 | 0.21 | -0.92 | 0.51 | 1.9 | 0.097 | 18.3 | 0.19 | -0.81 | 0.46 | 1.7 | 0.101 |
|  | L4 | 18.22 | 0.13 | -1.01 | 0.31 | 2 | 0.005 | 20.13 | 0.23 | 0.34 | 0.53 | -1.3 | 0.524 | 18.35 | 0.19 | -0.19 | 0.46 | 1.1 | 0.691 |
| *Overall p value* | |  |  |  |  |  | *0.007* |  |  |  |  |  | *0.202* |  |  |  |  |  | *0.232* |
|  |  | **ΔCT** | **s.e.m** | **ΔΔCT** | **s.e.m** | **FC** | **p** | **ΔCT** | **s.e.m** | **ΔΔCT** | **s.e.m** | **FC** | **p** | **ΔCT** | | **s.e.m** | **ΔΔCT** | **s.e.m** | **FC** |
|  |  | **IL1B** |  |  |  |  |  | **IL6** |  |  |  |  |  | **CD206** | |  |  |  |  |
| **M0/HT29** | L3 | 14.57 | 0.14 | -3.35 | 0.35 | 10.2 | <0.001 | 27.4 | 0.35 | -2.76 | 0.78 | 6.8 | 0.003 | 25.31 | 0.26 | 1.45 | 0.64 | -2.7 | 0.037 |
|  | L4 | 18.16 | 0.14 | -0.84 | 0.35 | 1.8 | 0.03 | 25.41 | 0.31 | -1.68 | 0.77 | 3.2 | 0.046 | 27.36 | 0.26 | 1.08 | 0.64 | -2.1 | 0.11 |
| *Overall p value* | |  |  |  |  |  | ***<0.001*** |  |  |  |  |  | ***0.004*** |  |  |  |  |  | ***0.038*** |
| **M0/SW480** | L3 | 14.46 | 0.3 | -3.22 | 0.66 | 9.3 | <0.001 | 23.14 | 0.49 | -3.74 | 1.1 | 13.4 | 0.005 | 24.52 | 0.35 | 0.43 | 0.87 | -1.3 | 0.625 |
|  | L4 | 12.1 | 0.3 | -1.14 | 0.66 | 2.2 | 0.111 | 27.88 | 0.49 | 2.03 | 1.1 | -4.1 | 0.09 | 26.77 | 0.35 | 2.1 | 0.87 | -4.3 | 0.028 |
| *Overall p value* | |  |  |  |  |  | ***<0.001*** |  |  |  |  |  | ***<0.001*** |  |  |  |  |  | *0.075* |
| **M2/HT29** | L3 | 16.38 | 0.23 | -3.23 | 0.56 | 9.4 | <0.001 | 24.96 | 0.29 | -3.8 | 0.71 | 14 | <0.001 | 19.67 | 0.21 | -0.87 | 0.53 | 1.8 | 0.115 |
|  | L4 | 16.22 | 0.23 | -3 | 0.56 | 8 | <0.001 | 25.79 | 0.37 | -1.76 | 0.74 | 3.4 | 0.036 | 20.29 | 0.21 | 0.78 | 0.53 | -1.7 | 0.156 |
| *Overall p value* | |  |  |  |  |  | ***<0.001*** |  |  |  |  |  | *0.008* |  |  |  |  |  | *0.114* |
| **M2/SW480** | L3 | 16.42 | 0.3 | -3.86 | 0.73 | 14.5 | <0.001 | 25.63 | 0.53 | -3.31 | 1.3 | 9.9 | 0.022 | 19.22 | 0.12 | -1.69 | 0.28 | 3.2 | <0.001 |
|  | L4 | 16.83 | 0.3 | -4.37 | 0.73 | 20.6 | <0.001 | 26.88 | 0.53 | -1.37 | 1.3 | 2.6 | 0.307 | 19.92 | 0.12 | 0.92 | 0.28 | -1.9 | 0.005 |
| *Overall p value* | |  |  |  |  |  | ***<0.001*** |  |  |  |  |  | ***0.045*** |  |  |  |  |  | *<0.001* |

**Supplementary Table 4. Macrophage Gene Expression following Adiponectin Treatment.** Macrophage gene expression following adiponectin treatment with CC cell line HT29 vs SW480 co-culture. s.e.m: standard error of the mean, FC: fold change, L3:6-hour treatment, L4: 18-hour treatment. Boldface reflects overall significant change in macrophage gene expression in co-culture with both CC cell lines. (P<0.05).

|  | | | | | | | | | | | | | | | | | | | |
| --- | --- | --- | --- | --- | --- | --- | --- | --- | --- | --- | --- | --- | --- | --- | --- | --- | --- | --- | --- |
| Cell lines/  Treatment length | | **ΔCT** | **s.e.m** | **ΔΔCT** | **s.e.m** | **FC** | **p** | **ΔCT** | **s.e.m** | **ΔΔCT** | **s.e.m** | **FC** | **p** | **ΔCT** | **s.e.m** | **ΔΔCT** | **s.e.m** | **FC** | **p** |
|  |  | **IRG1** |  |  |  |  |  | **CD80** |  |  |  |  |  | **CXCL10** | |  |  |  |  |
| **M0/HT29** | L3 | 24.16 | 0.74 | -1.63 | 1.82 | 3.1 | 0.385 | 16.78 | 0.17 | -3.5 | 0.37 | 11.3 | <0.001 | 20.89 | 0.18 | -0.54 | 0.4 | 1.4 | 0.201 |
|  | L4 | 27.73 | 0.83 | -3.65 | 1.85 | 12.6 | 0.069 | 18.4 | 0.15 | -1.23 | 0.36 | 2.4 | 0.004 | 20.97 | 0.16 | 0.41 | 0.39 | -1.3 | 0.318 |
| *Overall p value* | |  |  |  |  |  | 0.133 |  |  |  |  |  | <0.001 |  |  |  |  |  | *0.271* |
| **M0/SW480** | L3 | 25.43 | 0.34 | -7.37 | 0.84 | 165.3 | <0.001 | 19.85 | 0.25 | -1.29 | 0.61 | 2.4 | 0.053 | 23.22 | 0.29 | -1.45 | 0.72 | 2.7 | 0.066 |
|  | L4 | 25.72 | 0.34 | 1 | 0.84 | -2 | 0.252 | 23.82 | 0.28 | -0.73 | 0.62 | 1.7 | 0.262 | 24.75 | 0.38 | -2.82 | 0.76 | 7.1 | 0.003 |
| *Overall p value* | |  |  |  |  |  | <0.001 |  |  |  |  |  | 0.087 |  |  |  |  |  | *0.004* |
| **M2/HT29** | L3 | 21.66 | 0.33 | -3.8 | 0.8 | 14 | <0.001 | 21.13 | 0.12 | -0.77 | 0.29 | 1.7 | 0.018 | 13.01 | 0.41 | -6.03 | 0.91 | 65.1 | <0.001 |
|  | L4 | 23.81 | 0.33 | -2.65 | 0.8 | 6.3 | 0.004 | 21.32 | 0.12 | -0.04 | 0.29 | 1 | 0.881 | 17.03 | 0.41 | -0.32 | 0.91 | 1.2 | 0.73 |
| *Overall p value* | |  |  |  |  |  | ***<0.001*** |  |  |  |  |  | *0.056* |  |  |  |  |  | *<0.001* |
| **M2/SW480** | L3 | 27.18 | 0.3 | -1.87 | 0.68 | 3.7 | 0.017 | 20.29 | 0.3 | -0.18 | 0.74 | 1.1 | 0.816 | 22.64 | 0.33 | -0.36 | 0.74 | 1.3 | 0.629 |
|  | L4 | 27.75 | 0.3 | 1.03 | 0.68 | -2 | 0.153 | 20.31 | 0.3 | -0.46 | 0.74 | 1.4 | 0.547 | 23.63 | 0.29 | -0.59 | 0.72 | 1.5 | 0.43 |
| *Overall p value* | |  |  |  |  |  | ***0.027*** |  |  |  |  |  | *0.807* |  |  |  |  |  | *0.645* |
|  |  | **IL8** |  |  |  |  |  | **NFkB** |  |  |  |  |  | **TNF** |  |  |  |  |  |
| **M0/HT29** | L3 | 12.29 | 0.12 | -0.55 | 0.28 | 1.5 | 0.072 | 16.19 | 0.12 | -0.62 | 0.29 | 1.5 | 0.047 | 19.22 | 0.2 | -0.05 | 0.49 | 1 | 0.914 |
|  | L4 | 13.41 | 0.12 | -0.61 | 0.28 | 1.5 | 0.047 | 16.5 | 0.12 | 0 | 0.29 | -1 | 0.999 | 18.47 | 0.2 | 0.08 | 0.49 | -1.1 | 0.867 |
| *Overall p value* | |  |  |  |  |  | *0.035* |  |  |  |  |  | *0.13* |  |  |  |  |  | *0.98* |
| **M0/SW480** | L3 | 12.31 | 0.38 | -0.96 | 0.93 | 2 | 0.316 | 15.1 | 0.32 | -0.71 | 0.77 | 1.6 | 0.375 | 18.91 | 0.24 | -0.82 | 0.59 | 1.8 | 0.181 |
|  | L4 | 13 | 0.38 | 0.08 | 0.93 | -1.1 | 0.936 | 16.76 | 0.32 | -0.08 | 0.77 | 1.1 | 0.918 | 18.89 | 0.24 | 0.8 | 0.59 | -1.7 | 0.188 |
| *Overall p value* | |  |  |  |  |  | *0.593* |  |  |  |  |  | *0.663* |  |  |  |  |  | *0.178* |
| **M2/HT29** | L3 | 12.27 | 0.22 | -5.66 | 0.49 | 50.6 | <0.001 | 14.75 | 0.19 | -2.95 | 0.42 | 7.7 | <0.001 | 17.99 | 0.47 | -1.25 | 0.94 | 2.4 | 0.208 |
|  | L4 | 13.22 | 0.2 | -4.83 | 0.48 | 28.4 | <0.001 | 16.16 | 0.17 | -1.01 | 0.41 | 2 | 0.028 | 18.84 | 0.36 | 0.25 | 0.89 | -1.2 | 0.781 |
| *Overall p value* | |  |  |  |  |  | ***<0.001*** |  |  |  |  |  | ***<0.001*** |  |  |  |  |  | *0.422* |
| **M2/SW480** | L3 | 16.53 | 0.29 | -0.92 | 0.7 | 1.9 | 0.208 | 16.59 | 0.17 | -0.94 | 0.41 | 1.9 | 0.035 | 18.18 | 0.28 | 0.24 | 0.68 | -1.2 | 0.729 |
|  | L4 | 16.7 | 0.29 | -1.84 | 0.7 | 3.6 | 0.018 | 16.69 | 0.17 | -0.33 | 0.41 | 1.3 | 0.431 | 19.4 | 0.28 | -0.53 | 0.68 | 1.4 | 0.443 |
| *Overall p value* | |  |  |  |  |  | ***0.032*** |  |  |  |  |  | ***<0.001*** |  |  |  |  |  | *0.695* |
|  |  | **PPARG** | |  |  |  |  | **IL10** |  |  |  |  |  | **CCL22** |  |  |  |  |  |
| **M0/HT29** | L3 | 17.03 | 0.09 | -0.18 | 0.23 | 1.1 | 0.446 | 22.98 | 0.24 | -0.46 | 0.58 | 1.4 | 0.44 | 18.5 | 0.15 | -0.55 | 0.36 | 1.5 | 0.142 |
|  | L4 | 17.4 | 0.09 | 0.2 | 0.23 | -1.2 | 0.385 | 21.63 | 0.24 | -0.5 | 0.58 | 1.4 | 0.399 | 18.64 | 0.15 | -0.23 | 0.36 | 1.2 | 0.533 |
| *Overall p value* | |  |  |  |  |  | *0.51* |  |  |  |  |  | *0.516* |  |  |  |  |  | *0.277* |
| **M0/SW480** | L3 | 15.64 | 0.21 | -0.26 | 0.51 | 1.2 | 0.623 | 20.38 | 0.21 | 0.26 | 0.5 | -1.2 | 0.619 | 16.45 | 0.33 | -0.31 | 0.8 | 1.2 | 0.705 |
|  | L4 | 18.21 | 0.23 | 0.15 | 0.52 | -1.1 | 0.775 | 23.25 | 0.23 | -0.94 | 0.51 | 1.9 | 0.089 | 19.7 | 0.33 | -0.55 | 0.8 | 1.5 | 0.505 |
| *Overall p value* | |  |  |  |  |  | *0.846* |  |  |  |  |  | *0.202* |  |  |  |  |  | *0.74* |
| **M2/HT29** | L3 | 18.39 | 0.13 | -0.05 | 0.32 | 1 | 0.872 | 18.92 | 0.26 | -0.31 | 0.59 | 1.2 | 0.604 | 18.38 | 0.26 | -0.98 | 0.59 | 2 | 0.119 |
|  | L4 | 18.8 | 0.13 | 0.39 | 0.32 | -1.3 | 0.237 | 18.84 | 0.23 | 0.9 | 0.57 | -1.9 | 0.139 | 17.56 | 0.24 | -0.45 | 0.58 | 1.4 | 0.445 |
| *Overall p value* | |  |  |  |  |  | *0.48* |  |  |  |  |  | *0.286* |  |  |  |  |  | *0.221* |
| **M2/SW480** | L3 | 18.47 | 0.16 | 0.18 | 0.38 | -1.1 | 0.638 | 18.3 | 0.18 | 0.15 | 0.45 | -1.1 | 0.745 | 19.91 | 0.29 | 1.29 | 0.71 | -2.4 | 0.088 |
|  | L4 | 17.98 | 0.16 | -0.42 | 0.38 | 1.3 | 0.284 | 18.73 | 0.18 | 0.17 | 0.45 | -1.1 | 0.713 | 18.57 | 0.29 | -1.2 | 0.71 | 2.3 | 0.11 |
| *Overall p value* | |  |  |  |  |  | *0.498* |  |  |  |  |  | *0.883* |  |  |  |  |  | *0.074* |
|  |  | **ΔCT** | **s.e.m** | **ΔΔCT** | **s.e.m** | **FC** | **p** | **ΔCT** | **s.e.m** | **ΔΔCT** | **s.e.m** | **FC** | **p** | **ΔCT** | | **s.e.m** | **ΔΔCT** | **s.e.m** | **FC** |
|  |  | **IL1B** |  |  |  |  |  | **IL6** |  |  |  |  |  | **CD206** | |  |  |  |  |
| **M0/HT29** | L3 | 16 | 0.13 | 0.37 | 0.31 | -1.3 | 0.248 | 27.16 | 0.26 | -0.85 | 0.58 | 1.8 | 0.163 | 25.32 | 0.18 | -0.95 | 0.44 | 1.9 | 0.046 |
|  | L4 | 15.96 | 0.13 | -0.25 | 0.31 | 1.2 | 0.432 | 24.97 | 0.23 | -0.25 | 0.57 | 1.2 | 0.665 | 25.1 | 0.18 | 0.46 | 0.44 | -1.4 | 0.31 |
| *Overall p value* | |  |  |  |  |  | *0.375* |  |  |  |  |  | *0.336* |  |  |  |  |  | *0.085* |
| **M0/SW480** | L3 | 15.79 | 0.2 | -0.34 | 0.5 | 1.3 | 0.507 | 24.74 | 0.65 | 0.12 | 1.3 | -1.1 | 0.93 | 23.12 | 0.47 | -1.27 | 1.15 | 2.4 | 0.288 |
|  | L4 | 14.67 | 0.2 | 1.94 | 0.5 | -3.8 | 0.001 | 26.78 | 0.65 | -0.83 | 1.3 | 1.8 | 0.54 | 25.86 | 0.52 | 2.11 | 1.17 | -4.3 | 0.093 |
| *Overall p value* | |  |  |  |  |  | *0.004* |  |  |  |  |  | *0.816* |  |  |  |  |  | *0.144* |
| **M2/HT29** | L3 | 14.44 | 0.22 | -4.37 | 0.48 | 20.7 | <0.001 | 23.84 | 0.29 | -5.21 | 0.65 | 37 | <0.001 | 20.02 | 0.21 | -0.06 | 0.46 | 1 | 0.896 |
|  | L4 | 15.11 | 0.19 | -2.43 | 0.47 | 5.4 | <0.001 | 25.71 | 0.34 | -1.81 | 0.67 | 3.5 | 0.023 | 20.64 | 0.18 | 1.87 | 0.45 | -3.7 | 0.001 |
| *Overall p value* | |  |  |  |  |  | *<0.001* |  |  |  |  |  | *<0.001* |  |  |  |  |  | *0.004* |
| **M2/SW480** | L3 | 18.81 | 0.25 | 1.6 | 0.57 | -3 | 0.014 | 27.97 | 0.32 | -0.89 | 0.72 | 1.9 | 0.241 | 18.78 | 0.19 | 0.23 | 0.46 | -1.2 | 0.614 |
|  | L4 | 19.16 | 0.23 | -0.74 | 0.55 | 1.7 | 0.202 | 26.45 | 0.32 | -1.24 | 0.72 | 2.4 | 0.112 | 18.31 | 0.19 | -0.94 | 0.46 | 1.9 | 0.057 |
| *Overall p value* | |  |  |  |  |  | *0.025* |  |  |  |  |  | *0.15* |  |  |  |  |  | *0.138* |

**Supplementary Table 5. Macrophage Gene Expression following Leptin Treatment.** Macrophage gene expression following leptin treatment with CC cell line HT29 vs SW480 co-culture. s.e.m: standard error of the mean, FC: fold change, L3:6-hour treatment, L4: 18-hour treatment. Boldface reflects overall significant change in macrophage gene expression in co-culture with both CC cell lines. (P<0.05).

|  | | | | | | | | | | | | | | | | | | | |
| --- | --- | --- | --- | --- | --- | --- | --- | --- | --- | --- | --- | --- | --- | --- | --- | --- | --- | --- | --- |
| Cell lines/  Treatment length | | **ΔCT** | **s.e.m** | **ΔΔCT** | **s.e.m** | **FC** | **p** | **ΔCT** | **s.e.m** | **ΔΔCT** | **s.e.m** | **FC** | **p** | **ΔCT** | **s.e.m** | **ΔΔCT** | **s.e.m** | **FC** | **p** |
|  |  | **IRG1** |  |  |  |  |  | **CD80** |  |  |  |  |  | **CXCL10** | |  |  |  |  |
| **M0/HT29** | L3 | 26.29 | 0.57 | 2.55 | 1.26 | -5.9 | 0.09 | n/e |  |  |  |  |  | 25.33 | 0.84 | 1.44 | 1.88 | -2.7 | 0.473 |
|  | L4**^a^** | **-** | **-** | **-** | **-** | **-** | **-** | **-** | **-** | **-** | **-** | **-** | **-** | **-** | **-** | **-** | **-** | **-** | **-** |
| *Overall p value* | |  |  |  |  |  | *0.09* |  |  |  |  |  |  |  |  |  |  |  | *0.473* |
| **M0/SW480** | L3 | 29.29 | 0.43 | -0.93 | 0.86 | 1.9 | 0.299 | 22.54 | 0.34 | 0.87 | 0.84 | -1.8 | 0.319 | 27.05 | 0.87 | 4.75 | 1.74 | -26.9 | 0.026 |
|  | L4 | 30.53 | 0.33 | 1.49 | 0.81 | -2.8 | 0.093 | 26.96 | 0.38 | 4.13 | 0.86 | -17.5 | <0.001 | 26.1 | 0.87 | 4.86 | 1.74 | -29 | 0.023 |
| *Overall p value* | |  |  |  |  |  | *0.147* |  |  |  |  |  | *0.001* |  |  |  |  |  | *0.014* |
| **M2/HT29** | L3 | n/e |  |  |  |  |  | 22.79 | 0.34 | 0.91 | 0.84 | -1.9 | 0.298 | 18.98 | 0.47 | 1.93 | 1.04 | -3.8 | 0.084 |
|  | L4 | n/e |  |  |  |  |  | 25.01 | 0.38 | 3.38 | 0.86 | -10.4 | 0.001 | 19.17 | 0.42 | 2.36 | 1.02 | -5.1 | 0.036 |
| *Overall p value* | |  |  |  |  |  |  |  |  |  |  |  | ***0.004*** |  |  |  |  |  | ***0.033*** |
| **M2/SW480** | L3 | n/e |  |  |  |  |  | 23.67 | 0.43 | 2.57 | 1.06 | -5.9 | 0.041 | 26.72 | 0.79 | 6.12 | 1.78 | -69.4 | 0.014 |
|  | L4 **^a^** | **-** | **-** | **-** | **-** | **-** | **-** | **-** | **-** | **-** | **-** | **-** | **-** | **-** | **-** | **-** | **-** | **-** | **-** |
| *Overall p value* | |  |  |  |  |  |  |  |  |  |  |  | ***0.041*** |  |  |  |  |  | ***0.014*** |
|  |  | **IL8** |  |  |  |  |  | **NFkB** |  |  |  |  |  | **TNF** |  |  |  |  |  |
| **M0/HT29** | L3 | 25.28 | 0.75 | -0.78 | 1.83 | 1.7 | 0.682 | 20.24 | 1.06 | 3.86 | 2.6 | -14.5 | 0.176 | 15.34 | 0.25 | 0.67 | 0.56 | -1.6 | 0.278 |
|  | L4 **^a^** | **-** | **-** | **-** | **-** | **-** | **-** | **-** | **-** | **-** | **-** | **-** | **-** | **-** | **-** | **-** | **-** | **-** | **-** |
| *Overall p value* | |  |  |  |  |  | *0.682* |  |  |  |  |  | *0.176* |  |  |  |  |  | *0.278* |
| **M0/SW480** | L3 | 15.53 | 0.43 | 0.38 | 1.06 | -1.30 | 0.721 | 19.45 | 0.34 | 3.30 | 0.84 | -9.8 | 0.001 | 19.73 | 0.45 | -0.31 | 1.11 | 1.2 | 0.782 |
|  | L4 | 15.44 | 0.43 | -0.34 | 1.06 | 1.30 | 0.753 | 20.20 | 0.34 | 4.04 | 0.84 | -16.4 | <0.001 | 19.48 | 0.45 | 0.42 | 1.11 | -1.3 | 0.711 |
| *Overall p value* | |  |  |  |  |  | *0.89* |  |  |  |  |  | *<0.001* |  |  |  |  |  | *0.896* |
| **M2/HT29** | L3 | 14.96 | 0.22 | -3.42 | 0.54 | 10.7 | <0.001 | 17.35 | 0.12 | 0.71 | 0.3 | -1.6 | 0.034 | 22.95 | 0.72 | 3.68 | 1.6 | -12.8 | 0.038 |
|  | L4 | 12.79 | 0.22 | -7.61 | 0.54 | 195.3 | <0.001 | 17.46 | 0.14 | 1.33 | 0.31 | -2.5 | 0.001 | 24.39 | 0.64 | 7.05 | 1.57 | -132.7 | 0.001 |
| *Overall p value* | |  |  |  |  |  | *<0.001* |  |  |  |  |  | *0.001* |  |  |  |  |  | ***0.001*** |
| **M2/SW480** | L3 | 17.74 | 0.61 | 2.54 | 1.35 | -5.8 | 0.11 | 20.23 | 0.66 | 2.98 | 1.61 | -7.9 | 0.102 | 18.99 | 0.22 | 1.77 | 0.5 | -3.4 | 0.012 |
|  | L4 **^a^** | **-** | **-** | **-** | **-** | **-** | **-** | **-** | **-** | **-** | **-** | **-** | **-** | **-** | **-** | **-** | **-** | **-** | **-** |
| *Overall p value* | |  |  |  |  |  | *0.11* |  |  |  |  |  | *0.102* |  |  |  |  |  | ***0.012*** |
|  |  | **PPARG** | |  |  |  |  | **IL10** |  |  |  |  |  | **CCL22** |  |  |  |  |  |
| **M0/HT29** | L3 | 20.91 | 0.30 | 2.59 | 0.67 | -6 | 0.008 | 24.61 | 0.54 | -0.24 | 1.32 | 1.2 | 0.86 | 21.74 | 0.29 | 2.72 | 0.71 | -6.6 | 0.005 |
|  | L4 **^a^** | **-** | **-** | **-** | **-** | **-** | **-** | **-** | **-** | **-** | **-** | **-** | **-** | **-** | **-** | **-** | **-** | **-** | **-** |
| *Overall p value* | |  |  |  |  |  | ***0.008*** |  |  |  |  |  | *0.86* |  |  |  |  |  | ***0.005*** |
| **M0/SW480** | L3 | 19.83 | 0.44 | 3.08 | 1.08 | -8.5 | 0.011 | 25.68 | 0.49 | 4.44 | 1.20 | -21.8 | 0.002 | 21.94 | 0.60 | 3.35 | 1.48 | -10.2 | 0.04 |
|  | L4 | 21.22 | 0.44 | 4.83 | 1.08 | -28.4 | <0.001 | 26.78 | 0.55 | 5.30 | 1.22 | -39.5 | 0.001 | 23.81 | 0.68 | 6.02 | 1.51 | -65 | 0.001 |
| *Overall p value* | |  |  |  |  |  | ***<0.001*** |  |  |  |  |  | *<0.001* |  |  |  |  |  | ***0.002*** |
| **M2/HT29** | L3 | 20.27 | 0.19 | 1.27 | 0.47 | -2.4 | 0.017 | 19.97 | 0.17 | 0.51 | 0.41 | -1.4 | 0.233 | 21.37 | 0.15 | 2.05 | 0.37 | -4.2 | <0.001 |
|  | L4 | 18.61 | 0.21 | -0.01 | 0.48 | 1 | 0.984 | 19.78 | 0.17 | 1.57 | 0.41 | -3 | 0.001 | 21.14 | 0.15 | 2.75 | 0.37 | -6.7 | <0.001 |
| *Overall p value* | |  |  |  |  |  | *0.053* |  |  |  |  |  | ***0.004*** |  |  |  |  |  | *<0.001* |
| **M2/SW480** | L3 | 21.87 | 0.61 | 2.17 | 1.49 | -4.5 | 0.182 | 23.42 | 0.49 | 3.85 | 1.10 | -14.4 | 0.013 | 20.81 | 0.53 | 1.62 | 1.30 | -3.1 | 0.249 |
|  | L4 **^a^** | **-** | **-** | **-** | **-** | **-** | **-** | **-** | **-** | **-** | **-** | **-** | **-** | **-** | **-** | **-** | **-** | **-** | **-** |
| *Overall p value* | |  |  |  |  |  | 0.182 |  |  |  |  |  | **0.013** |  |  |  |  |  | 0.249 |
|  |  | **ΔCT** | **s.e.m** | **ΔΔCT** | **s.e.m** | **FC** | **p** | **ΔCT** | **s.e.m** | **ΔΔCT** | **s.e.m** | **FC** | **p** | **ΔCT** | **s.e.m** | **ΔΔCT** | **s.e.m** | **FC** | **p** |
|  |  | **IL1B** |  |  |  |  |  | **IL6** |  |  |  |  |  | **CD206** | |  |  |  |  |
| **M0/HT29** | L3 | 13.11 | 0.21 | 2.05 | 0.46 | -4.2 | 0.004 | n/e |  |  |  |  |  | 26.27 | 0.41 | 2.54 | 1 | -5.8 | 0.035 |
|  | L4 **^a^** | **-** | **-** | **-** | **-** | **-** | **-** | **-** | **-** | **-** | **-** | **-** | **-** | **-** | **-** | **-** | **-** | **-** | **-** |
| *Overall p value* | |  |  |  |  |  | *0.004* |  |  |  |  |  |  |  |  |  |  |  | ***0.035*** |
| **M0/SW480** | L3 | 18.57 | 0.56 | -0.31 | 1.36 | 1.2 | 0.822 | n/e |  |  |  |  |  | 27.45 | 0.37 | 2.44 | 0.92 | -5.4 | 0.017 |
|  | L4 | 15.60 | 0.56 | -1.62 | 1.36 | 3.1 | 0.253 | 27.52 | 0.43 | 1.2 | 1.06 | -2.3 | 0.291 | 27.34 | 0.37 | 3.62 | 0.92 | -12.3 | 0.001 |
| *Overall p value* | |  |  |  |  |  | *0.498* |  |  |  |  |  | *0.291* |  |  |  |  |  | ***0.001*** |
| **M2/HT29** | L3 | 20.33 | 0.25 | 0.59 | 0.60 | -1.5 | 0.346 | 29.57 | 0.23 | -0.37 | 0.56 | 1.3 | 0.516 | 20.60 | 0.18 | 1.24 | 0.44 | -2.4 | 0.014 |
|  | L4 | 19.51 | 0.25 | -0.22 | 0.60 | 1.2 | 0.716 | 28.68 | 0.23 | 2.12 | 0.56 | -4.3 | 0.002 | 20.46 | 0.20 | 2.00 | 0.45 | -4 | 0.001 |
| *Overall p value* | |  |  |  |  |  | *0.593* |  |  |  |  |  | ***0.005*** |  |  |  |  |  | *<0.001* |
| **M2/SW480** | L3 | 20.79 | 0.4 | 2.1 | 0.89 | -4.3 | 0.057 | 27.74 | 0.38 | 2.23 | 0.84 | -4.7 | 0.039 | 21.66 | 0.65 | 1.85 | 1.45 | -3.6 | 0.248 |
|  | L4 **^a^** | **-** | **-** | **-** | **-** | **-** | **-** | **-** | **-** | **-** | **-** | **-** | **-** | **-** | **-** | **-** | **-** | **-** | **-** |
| *Overall p value* | |  |  |  |  |  | *0.057* |  |  |  |  |  | ***0.039*** |  |  |  |  |  | *0.248* |

**Supplementary Table 6. Macrophage Gene Expression following 4-Octyl-Itaconate (4OI) Treatment.** Macrophage gene expression following 4 octyl-itaconate treatment with CC cell line HT29 vs SW480 co-culture. s.e.m: standard error of the mean, FC: fold change, L3:6-hour treatment, L4: 18-hour treatment. Boldface reflects overall significant change in macrophage gene expression in co-culture with both CC cell lines (P<0.05). ^a^ 4OI caused macrophage death following 18 hours treatment in M0/HT29 and M2/SW480 therefore gene expression not measured. n/e: not expressed.

|  | | | | | | | | | | | | | | | | | | | |
| --- | --- | --- | --- | --- | --- | --- | --- | --- | --- | --- | --- | --- | --- | --- | --- | --- | --- | --- | --- |
| Cell lines/  Treatment length | | **ΔCT** | **s.e.m** | **ΔΔCT** | **s.e.m** | **FC** | **p** | **ΔCT** | **s.e.m** | **ΔΔCT** | **s.e.m** | **FC** | **p** | **ΔCT** | **s.e.m** | **ΔΔCT** | **s.e.m** | **FC** | **p** |
|  |  | **IRG1** |  |  |  |  |  | **CD80** |  |  |  |  |  | **CXCL10** | |  |  |  |  |
| **M0/HT29** | L3 | 24.81 | 0.52 | 1.53 | 1.16 | -2.9 | 0.238 | n/e |  |  |  |  |  | 23.23 | 0.68 | 0.67 | 1.66 | -1.6 | 0.691 |
|  | L4 | n/e |  |  |  |  |  | 23.14 | 0.16 | -0.54 | 0.4 | 1.5 | 0.219 | 28.06 | 0.68 | 5.35 | 1.66 | -40.8 | 0.005 |
| *Overall p value* | |  |  |  |  |  | *0.238* |  |  |  |  |  | *0.219* |  |  |  |  |  | ***0.017*** |
| **M0/SW480** | L3 | 30.38 | 0.56 | 2.34 | 1.12 | -5.1 | 0.059 | 21.35 | 0.55 | 0.01 | 1.34 | -1 | 0.992 | 24.85 | 0.28 | -0.64 | 0.69 | 1.6 | 0.367 |
|  | L4 | 30.36 | 0.44 | 3 | 1.07 | -8 | 0.016 | 25.19 | 0.55 | 0.53 | 1.34 | -1.4 | 0.7 | 29.66 | 0.28 | 5.90 | 0.69 | -59.5 | <0.001 |
| *Overall p value* | |  |  |  |  |  | *0.015* |  |  |  |  |  | *0.926* |  |  |  |  |  | ***<0.001*** |
| **M2/HT29** | L3 | n/e |  |  |  |  |  | 21.39 | 0.62 | 0.08 | 1.51 | -1.1 | 0.956 | 23.94 | 0.47 | -0.42 | 1.06 | 1.3 | 0.7 |
|  | L4 | 28.83 | 0.75 | -3.27 | 1.69 | 9.6 | 0.101 | 20.95 | 0.62 | -2.25 | 1.51 | 4.7 | 0.156 | 27.02 | 0.42 | 1.08 | 1.04 | -2.1 | 0.315 |
| *Overall p value* | |  |  |  |  |  | *0.101* |  |  |  |  |  | *0.354* |  |  |  |  |  | *0.552* |
| **M2/SW480** | L3 | 32.71 | 0.55 | 2.05 | 1.23 | -4.1 | 0.123 | 20.83 | 0.15 | -0.01 | 0.33 | 1 | 0.977 | 25.14 | 0.43 | 2.51 | 0.95 | -5.7 | 0.019 |
|  | L4 | 31.57 | 0.55 | 1.16 | 1.23 | -2.2 | 0.364 | 20.62 | 0.13 | -0.82 | 0.32 | 1.8 | 0.024 | 25.80 | 0.38 | 1.92 | 0.93 | -3.8 | 0.058 |
| *Overall p value* | |  |  |  |  |  | *0.204* |  |  |  |  |  | *0.07* |  |  |  |  |  | *0.016* |
|  |  | **IL8** |  |  |  |  |  | **NFkB** |  |  |  |  |  | **TNF** |  |  |  |  |  |
| **M0/HT29** | L3 | 11.53 | 0.53 | -0.77 | 1.29 | 1.7 | 0.558 | 16.56 | 0.36 | 0.31 | 0.89 | -1.2 | 0.731 | 19.04 | 0.42 | 1.16 | 0.94 | -2.2 | 0.234 |
|  | L4 | 13.04 | 0.53 | -4.18 | 1.29 | 18.1 | 0.005 | 17.66 | 0.36 | 1.14 | 0.89 | -2.2 | 0.219 | 18.49 | 0.37 | -1.51 | 0.92 | 2.9 | 0.122 |
| *Overall p value* | |  |  |  |  |  | ***0.016*** |  |  |  |  |  | *0.434* |  |  |  |  |  | *0.156* |
| **M0/SW480** | L3 | 11.71 | 0.15 | -3.03 | 0.36 | 8.2 | <0.001 | 16.65 | 0.1 | 0.34 | 0.24 | -1.3 | 0.186 | 19.12 | 0.24 | -1.19 | 0.58 | 2.3 | 0.055 |
|  | L4 | 10.19 | 0.15 | -1.91 | 0.36 | 3.8 | <0.001 | 18.34 | 0.1 | 1.22 | 0.24 | -2.3 | <0.001 | 18.98 | 0.24 | 0.75 | 0.58 | -1.7 | 0.213 |
| *Overall p value* | |  |  |  |  |  | ***<0.001*** |  |  |  |  |  | *<0.001* |  |  |  |  |  | *0.08* |
| **M2/HT29** | L3 | 13.76 | 0.31 | -5.28 | 0.75 | 38.9 | <0.001 | 17.09 | 0.4 | 0.14 | 0.99 | -1.1 | 0.889 | 16.15 | 0.49 | 0.52 | 1.2 | -1.4 | 0.669 |
|  | L4 | 14.02 | 0.31 | -5.99 | 0.75 | 63.7 | <0.001 | 18.47 | 0.4 | 0.98 | 0.99 | -2 | 0.337 | 17.76 | 0.49 | -3.24 | 1.2 | 9.4 | 0.016 |
| *Overall p value* | |  |  |  |  |  | ***<0.001*** |  |  |  |  |  | *0.616* |  |  |  |  |  | ***0.046*** |
| **M2/SW480** | L3 | 13.95 | 0.16 | -2.53 | 0.39 | 5.8 | <0.001 | 17.43 | 0.14 | 0.73 | 0.32 | -1.7 | 0.038 | 16.5 | 0.13 | -0.73 | 0.33 | 1.7 | 0.04 |
|  | L4 | 13.84 | 0.16 | -4.66 | 0.39 | 25.2 | <0.001 | 17.53 | 0.13 | 0.3 | 0.31 | -1.2 | 0.355 | 17.37 | 0.13 | -1.69 | 0.33 | 3.2 | <0.001 |
| *Overall p value* | |  |  |  |  |  | ***<0.001*** |  |  |  |  |  | *0.078* |  |  |  |  |  | ***<0.001*** |
|  |  | **PPARG** | |  |  |  |  | **IL10** |  |  |  |  |  | **CCL22** |  |  |  |  |  |
| **M0/HT29** | L3 | 18.99 | 0.46 | -0.57 | 1.13 | 1.5 | 0.619 | 23.94 | 0.53 | -1.07 | 1.29 | 2.1 | 0.419 | 19.86 | 0.5 | 0.38 | 1.22 | -1.3 | 0.763 |
|  | L4 | 16.99 | 0.46 | -0.07 | 1.13 | 1.1 | 0.948 | 21.18 | 0.53 | -0.98 | 1.29 | 2 | 0.459 | 18.76 | 0.5 | 0.99 | 1.22 | -2 | 0.429 |
| *Overall p value* | |  |  |  |  |  | *0.878* |  |  |  |  |  | *0.544* |  |  |  |  |  | *0.693* |
| **M0/SW480** | L3 | 16.46 | 0.14 | -0.46 | 0.35 | 1.4 | 0.203 | 21.32 | 0.22 | -0.46 | 0.48 | 1.4 | 0.357 | 18.78 | 0.17 | -0.15 | 0.42 | 1.1 | 0.725 |
|  | L4 | 18.52 | 0.14 | -0.14 | 0.35 | 1.1 | 0.689 | 19.68 | 0.19 | -3.37 | 0.47 | 10.4 | <0.001 | 21.18 | 0.17 | 0.9 | 0.42 | -1.9 | 0.047 |
| *Overall p value* | |  |  |  |  |  | *0.402* |  |  |  |  |  | *<0.001* |  |  |  |  |  | *0.124* |
| **M2/HT29** | L3 | 19.16 | 0.47 | 0.57 | 1.14 | -1.5 | 0.624 | 17.24 | 0.3 | -2.04 | 0.74 | 4.1 | 0.015 | 20.59 | 0.62 | 0.08 | 1.52 | -1.1 | 0.959 |
|  | L4 | 18.49 | 0.47 | 0.72 | 1.14 | -1.6 | 0.537 | 17.4 | 0.3 | -2.27 | 0.74 | 4.8 | 0.008 | 20.72 | 0.62 | -1.18 | 1.52 | 2.3 | 0.451 |
| *Overall p value* | |  |  |  |  |  | *0.728* |  |  |  |  |  | ***0.003*** |  |  |  |  |  | *0.745* |
| **M2/SW480** | L3 | 19.28 | 0.2 | -0.62 | 0.49 | 1.5 | 0.221 | 18.62 | 0.07 | -0.68 | 0.16 | 1.6 | 0.001 | 19.33 | 0.21 | 0.11 | 0.47 | -1.1 | 0.822 |
|  | L4 | 19.43 | 0.2 | -0.59 | 0.49 | 1.5 | 0.245 | 18.66 | 0.07 | -0.51 | 0.16 | 1.4 | 0.006 | 19.96 | 0.19 | 0.68 | 0.46 | -1.6 | 0.165 |
| *Overall p value* | |  |  |  |  |  | *0.245* |  |  |  |  |  | ***<0.001*** |  |  |  |  |  | *0.359* |
|  |  | **ΔCT** | **s.e.m** | **ΔΔCT** | **s.e.m** | **FC** | **p** | **ΔCT** | **s.e.m** | **ΔΔCT** | **s.e.m** | **FC** | **p** | **ΔCT** | | **s.e.m** | **ΔΔCT** | **s.e.m** | **FC** |
|  |  | **IL1B** |  |  |  |  |  | **IL6** |  |  |  |  |  | **CD206** | |  |  |  |  |
| **M0/HT29** | L3 | 11.41 | 0.46 | -0.14 | 1.12 | 1.1 | 0.904 | n/e |  |  |  |  |  | 22.74 | 0.45 | 0.51 | 1.1 | -1.4 | 0.653 |
|  | L4 | 16.96 | 0.46 | -2.73 | 1.12 | 6.6 | 0.027 | 28.85 | 0.56 | 2.15 | 1.25 | -4.5 | 0.135 | 24.16 | 0.45 | 1.2 | 1.1 | -2.3 | 0.294 |
| *Overall p value* | |  |  |  |  |  | *0.08* |  |  |  |  |  | *0.135* |  |  |  |  |  | *0.514* |
| **M0/SW480** | L3 | 17.24 | 0.24 | -1.57 | 0.54 | 3 | 0.011 | 28.14 | 0.62 | -0.26 | 1.52 | 1.2 | 0.867 | 26.11 | 0.28 | 1.29 | 0.62 | -2.4 | 0.059 |
|  | L4 | 14.05 | 0.22 | 1.23 | 0.53 | -2.3 | 0.035 | n/e |  |  |  |  |  | 25.66 | 0.25 | 0.73 | 0.61 | -1.7 | 0.256 |
| *Overall p value* | |  |  |  |  |  | *0.008* |  |  |  |  |  | *0.867* |  |  |  |  |  | *0.093* |
| **M2/HT29** | L3 | 17.47 | 0.61 | 2.18 | 1.36 | -4.5 | 0.133 | n/e |  |  |  |  |  | 20.91 | 0.61 | -0.3 | 1.5 | 1.2 | 0.846 |
|  | L4 | 17.62 | 0.55 | -3.58 | 1.34 | 11.9 | 0.018 | n/e |  |  |  |  |  | 20.74 | 0.61 | -1.67 | 1.5 | 3.2 | 0.285 |
| *Overall p value* | |  |  |  |  |  | ***0.025*** |  |  |  |  |  |  |  |  |  |  |  | *0.544* |
| **M2/SW480** | L3 | 19.65 | 0.11 | -0.36 | 0.26 | 1.3 | 0.187 | 28.07 | 0.47 | 0.22 | 1.15 | -1.2 | 0.85 | 19.23 | 0.21 | -0.47 | 0.47 | 1.4 | 0.334 |
|  | L4 | 19.46 | 0.11 | -2.07 | 0.26 | 4.2 | <0.001 | 27.93 | 0.53 | 0.99 | 1.18 | -2 | 0.412 | 19.42 | 0.19 | 0.71 | 0.46 | -1.6 | 0.145 |
| *Overall p value* | |  |  |  |  |  | ***<0.001*** |  |  |  |  |  | *0.694* |  |  |  |  |  | *0.22* |

**Supplementary Table 7. Macrophage Gene Expression following Dimethyl-itaconate (DI) Treatment.** Macrophage gene expression following dimethyl-itaconate treatment with CC cell line HT29 vs SW480 co-culture. s.e.m: standard error of the mean, FC: fold change, L3:6-hour treatment, L4: 18-hour treatment. Boldface reflects overall significant change in macrophage gene expression in co-culture with both CC cell lines (P<0.05). n/e: not expressed.

|  | | | | | | | | | | | | | | | | | | | |  |
| --- | --- | --- | --- | --- | --- | --- | --- | --- | --- | --- | --- | --- | --- | --- | --- | --- | --- | --- | --- | --- |
| Cell lines/  Treatment length | | **ΔCT** | **s.e.m** | **ΔΔCT** | **s.e.m** | **FC** | **p** | **ΔCT** | **s.e.m** | **ΔΔCT** | **s.e.m** | **FC** | **p** | **ΔCT** | **s.e.m** | **ΔΔCT** | **s.e.m** | **FC** | **p** |  |
|  |  | **ABCG8** | |  |  |  |  | **CCL22** |  |  |  |  |  | **CXCL10** | |  |  |  |  |  |
| **HT29/M0** | L3 | n/e |  |  |  |  |  | 25.1 | 0.24 | -0.39 | 0.54 | 1.3 | 0.485 | 26.49 | 0.26 | 0.6 | 0.63 | -1.5 | 0.356 |  |
|  | L4 | n/e |  |  |  |  |  | 27.6 | 0.28 | -0.51 | 0.56 | 1.4 | 0.382 | 27.02 | 0.33 | -0.75 | 0.66 | 1.7 | 0.277 |  |
| *Overall p value* | |  |  |  |  |  |  |  |  |  |  |  | *0.529* |  |  |  |  |  | *0.361* |  |
| **SW480/M0** | L3 | 26.32 | 0.45 | 0.05 | 1.1 | -1 | 0.967 | n/e |  |  |  |  |  | 26.78 | 0.43 | -3.48 | 0.97 | 11.2 | 0.011 |  |
|  | L4 | n/e |  |  |  |  |  | n/e |  |  |  |  |  | n/e |  |  |  |  |  |  |
| *Overall p value* | |  |  |  |  |  | *0.967* |  |  |  |  |  |  |  |  |  |  |  | ***0.011*** |  |
| **HT29/M2** | L3 | n/e |  |  |  |  |  | 27.34 | 0.62 | 1.45 | 1.24 | -2.7 | 0.267 | 23.31 | 0.64 | 0.49 | 1.57 | -1.4 | 0.756 |  |
|  | L4 | n/e |  |  |  |  |  | 26.31 | 0.48 | -1.37 | 1.18 | 2.6 | 0.267 | 24.86 | 0.64 | -0.66 | 1.57 | 1.6 | 0.681 |  |
| *Overall p value* | |  |  |  |  |  |  |  |  |  |  |  | *0.295* |  |  |  |  |  | *0.872* |  |
| **SW480/M2** | L3 | n/e |  |  |  |  |  | n/e |  |  |  |  |  | 25.51 | 0.23 | -3.26 | 0.56 | 9.6 | <0.001 |  |
|  | L4 | n/e |  |  |  |  |  | n/e |  |  |  |  |  | n/e |  |  |  |  |  |  |
| *Overall p value* | |  |  |  |  |  |  |  |  |  |  |  |  |  |  |  |  |  | ***<0.001*** |  |
|  |  | **GATA5** | |  |  |  |  | **IL10** |  |  |  |  |  | **IL1β** | |  |  |  |  |  |
| **HT29/M0** | L3 | 24.06 | 0.17 | -0.8 | 0.38 | 1.7 | 0.058 | n/e |  |  |  |  |  | 25.49 | 0.27 | 0.53 | 0.66 | -1.4 | 0.434 |  |
|  | L4 | 24.3 | 0.15 | 0.09 | 0.38 | -1.1 | 0.819 | n/e |  |  |  |  |  | 25.06 | 0.27 | -1.02 | 0.66 | 2 | 0.142 |  |
| *Overall p value* | |  |  |  |  |  | *0.152* |  |  |  |  |  |  |  |  |  |  |  | *0.25* |  |
| **SW480/M0** | L3 | n/e |  |  |  |  |  | 18.22 | 0.18 | 0.16 | 0.44 | -1.1 | 0.717 | n/e |  |  |  |  |  |  |
|  | L4 | n/e |  |  |  |  |  | 27.9 | 0.23 | 1.95 | 0.46 | -3.9 | 0.001 | 27.37 | 0.23 | -0.14 | 0.52 | 1.1 | 0.796 |  |
| *Overall p value* | |  |  |  |  |  |  |  |  |  |  |  | ***0.004*** |  |  |  |  |  | *0.796* |  |
| **HT29/M2** | L3 | 26.05 | 0.63 | 1.29 | 1.42 | -2.5 | 0.377 | n/e |  |  |  |  |  | 26.97 | 0.6 | 1.85 | 1.34 | -3.6 | 0.191 |  |
|  | L4 | 24.21 | 0.57 | 0.26 | 1.39 | -1.2 | 0.854 | n/e |  |  |  |  |  | 26.46 | 0.6 | 1.39 | 1.34 | -2.6 | 0.32 |  |
| *Overall p value* | |  |  |  |  |  | *0.657* |  |  |  |  |  |  |  |  |  |  |  | *0.262* |  |
| **SW480/M2** | L3 | n/e |  |  |  |  |  | 20.88 | 0.13 | 0.1 | 0.33 | -1.1 | 0.774 | 27.88 | 0.52 | 0.29 | 1.28 | -1.2 | 0.825 |  |
|  | L4 | n/e |  |  |  |  |  | 21.28 | 0.13 | -0.15 | 0.33 | 1.1 | 0.653 | n/e |  |  |  |  |  |  |
| *Overall p value* | |  |  |  |  |  |  |  |  |  |  |  | *0.864* |  |  |  |  |  | *0.825* |  |
|  |  | **IL6** | |  |  |  |  | **IL8** |  |  |  |  |  | **LCT** | |  |  |  |  |  |
| **HT29/M0** | L3 | n/e |  |  |  |  |  | 19.19 | 0.2 | -0.78 | 0.48 | 1.7 | 0.125 | n/e |  |  |  |  |  |  |
|  | L4 | n/e |  |  |  |  |  | 20.44 | 0.2 | -0.41 | 0.48 | 1.3 | 0.414 | n/e |  |  |  |  |  |  |
| *Overall p value* | |  |  |  |  |  |  |  |  |  |  |  | *0.221* |  |  |  |  |  |  |  |
| **SW480/M0** | L3 | n/e |  |  |  |  |  | 16.91 | 0.25 | -2.47 | 0.6 | 5.5 | 0.001 | 24.64 | 0.54 | 2.15 | 1.2 | -4.4 | 0.104 |  |
|  | L4 | 19.69 | 0.3 | -1.16 | 0.72 | 2.2 | 0.148 | 21.46 | 0.25 | 0.11 | 0.6 | -1.1 | 0.859 | 24.57 | 0.62 | -2.26 | 1.24 | 4.8 | 0.099 |  |
| *Overall p value* | |  |  |  |  |  | *0.148* |  |  |  |  |  | ***0.003*** |  |  |  |  |  | *0.081* |  |
| **HT29/M2** | L3 | n/e |  |  |  |  |  | 19.8 | 0.11 | -0.02 | 0.27 | 1 | 0.949 | n/e |  |  |  |  |  |  |
|  | L4 | n/e |  |  |  |  |  | 19.47 | 0.11 | -0.17 | 0.27 | 1.1 | 0.534 | n/e |  |  |  |  |  |  |
| *Overall p value* | |  |  |  |  |  |  |  |  |  |  |  | *0.818* |  |  |  |  |  |  |  |
| **SW480/M2** | L3 | n/e |  |  |  |  |  | 19.81 | 0.12 | -0.79 | 0.3 | 1.7 | 0.016 | 25.05 | 0.37 | -1.85 | 0.82 | 3.6 | 0.048 |  |
|  | L4 | n/e |  |  |  |  |  | 19.91 | 0.12 | 0.13 | 0.3 | -1.1 | 0.669 | 27.51 | 0.42 | -2.3 | 0.85 | 4.9 | 0.022 |  |
| *Overall p value* | |  |  |  |  |  |  |  |  |  |  |  | ***0.048*** |  |  |  |  |  | **0.018** |  |
|  |  | **ΔCT** | **s.e.m** | **ΔΔCT** | **s.e.m** | **FC** | **p** | **ΔCT** | **s.e.m** | **ΔΔCT** | **s.e.m** | **FC** | **p** | **ΔCT** | **s.e.m** | **ΔΔCT** | **s.e.m** | **FC** | **p** |  |
|  |  | **NFκB** | |  |  |  |  | **PLIN4** |  |  |  |  |  | **PPARα** | |  |  |  |  |  |
| **HT29/M0** | L3 | 16.73 | 0.25 | -0.28 | 0.56 | 1.2 | 0.626 | n/e |  |  |  |  |  | 20.26 | 0.22 | 0.13 | 0.54 | -1.1 | 0.817 |  |
|  | L4 | 19.06 | 0.22 | 1 | 0.54 | -2 | 0.088 | n/e |  |  |  |  |  | 20.25 | 0.22 | 0.36 | 0.54 | -1.3 | 0.514 |  |
| *Overall p value* | |  |  |  |  |  | *0.2* |  |  |  |  |  |  |  |  |  |  |  | *0.782* |  |
| **SW480/M0** | L3 | 15.16 | 0.2 | -0.53 | 0.5 | 1.4 | 0.32 | n/e |  |  |  |  |  | 21.99 | 0.2 | -0.1 | 0.49 | 1.1 | 0.841 |  |
|  | L4 | n/e |  |  |  |  |  | n/e |  |  |  |  |  | 22.69 | 0.2 | 0.48 | 0.49 | -1.4 | 0.349 |  |
| *Overall p value* | |  |  |  |  |  | *0.32* |  |  |  |  |  |  |  |  |  |  |  | *0.624* |  |
| **HT29/M2** | L3 | 16.63 | 0.1 | -0.62 | 0.24 | 1.5 | 0.019 | 23.58 | 0.24 | -0.63 | 0.59 | 1.6 | 0.295 | 20.07 | 0.39 | 0.09 | 0.96 | -1.1 | 0.925 |  |
|  | L4 | 17.5 | 0.1 | -0.16 | 0.24 | 1.1 | 0.509 | 21.45 | 0.24 | 0.39 | 0.59 | -1.3 | 0.511 | 20.36 | 0.44 | 0.12 | 0.98 | -1.1 | 0.9 |  |
| *Overall p value* | |  |  |  |  |  | *0.051* |  |  |  |  |  | *0.461* |  |  |  |  |  | *0.987* |  |
| **SW480/M2** | L3 | 16.39 | 0.2 | -0.61 | 0.5 | 1.5 | 0.24 | 23.16 | 0.13 | -0.11 | 0.32 | 1.1 | 0.741 | 22.79 | 0.16 | 0.07 | 0.4 | -1.1 | 0.863 |  |
|  | L4 | 16.97 | 0.2 | -0.21 | 0.5 | 1.2 | 0.675 | 23.81 | 0.13 | 0.42 | 0.32 | -1.3 | 0.208 | 21.69 | 0.16 | -0.02 | 0.4 | 1 | 0.97 |  |
| *Overall p value* | |  |  |  |  |  | *0.451* |  |  |  |  |  | *0.42* |  |  |  |  |  | *0.984* |  |
|  |  | **PPARγ** | |  |  |  |  | **SERPINE** | |  |  |  |  | **TNFα** | |  |  |  |  |  |
| **HT29/M0** | L3 | 16.78 | 0.25 | -0.09 | 0.61 | 1.1 | 0.887 | 22.83 | 0.17 | -0.17 | 0.41 | 1.1 | 0.683 | 27.49 | 0.48 | 0.08 | 1.08 | -1.1 | 0.94 |  |
|  | L4 | 16.35 | 0.25 | -0.03 | 0.61 | 1 | 0.963 | 23.01 | 0.17 | -0.66 | 0.41 | 1.6 | 0.127 | 27.36 | 0.43 | -2.79 | 1.05 | 6.9 | 0.019 |  |
| *Overall p value* | |  |  |  |  |  | *0.989* |  |  |  |  |  | *0.279* |  |  |  |  |  | *0.058* |  |
| **SW480/M0** | L3 | 18.15 | 0.2 | 0.42 | 0.49 | -1.3 | 0.41 | 15.52 | 0.34 | -1.79 | 0.84 | 3.5 | 0.049 | 20.68 | 0.18 | -4.61 | 0.45 | 24.5 | <0.001 |  |
|  | L4 | 23.39 | 0.26 | -3.08 | 0.52 | 8.4 | <0.001 | 17.81 | 0.34 | -0.23 | 0.84 | 1.2 | 0.791 | n/e |  |  |  |  |  |  |
| *Overall p value* | |  |  |  |  |  | ***<0.001*** |  |  |  |  |  | *0.132* |  |  |  |  |  | ***<0.001*** |  |
| **HT29/M2** | L3 | 16.47 | 0.13 | -0.05 | 0.31 | 1 | 0.879 | 22.5 | 0.39 | 0.07 | 0.96 | -1.1 | 0.94 | 25.86 | 0.48 | 0.05 | 1.17 | -1 | 0.966 |  |
|  | L4 | 16.89 | 0.13 | -0.21 | 0.31 | 1.2 | 0.509 | 22.15 | 0.39 | -0.35 | 0.96 | 1.3 | 0.723 | 24.82 | 0.48 | 0.1 | 1.17 | -1.1 | 0.931 |  |
| *Overall p value* | |  |  |  |  |  | *0.789* |  |  |  |  |  | *0.934* |  |  |  |  |  | *0.995* |  |
| **SW480/M2** | L3 | 20.42 | 0.14 | -0.22 | 0.34 | 1.2 | 0.519 | 17.41 | 0.14 | -0.86 | 0.33 | 1.8 | 0.02 | 23.75 | 0.33 | -5.01 | 0.8 | 32.2 | <0.001 |  |
|  | L4 | 20.39 | 0.14 | 0.02 | 0.34 | -1 | 0.96 | 16.81 | 0.14 | 0.33 | 0.33 | -1.3 | 0.338 | 25.93 | 0.33 | -2.55 | 0.8 | 5.9 | 0.006 |  |
| *Overall p value* | |  |  |  |  |  | *0.806* |  |  |  |  |  | ***0.044*** |  |  |  |  |  | ***<0.001*** |  |

**Supplementary Table 8. Cancer Cell Line Gene Expression Following Adiponectin Treatment.** Cancer cell line gene expression following adiponectin treatment with M0 or M2 macrophage co-culture. s.e.m: standard error of the mean, FC: fold change, L3:6-hour treatment, L4: 18-hour treatment. n/e: not expressed. Boldface reflects overall significant change in gene expression (p<0.05). *CD206 not expressed in all experimental conditions (data not shown).*

|  | | | | | | | | | | | | | | | | | | | |  |
| --- | --- | --- | --- | --- | --- | --- | --- | --- | --- | --- | --- | --- | --- | --- | --- | --- | --- | --- | --- | --- |
| Cell lines/  Treatment length | | **ΔCT** | **s.e.m** | **ΔΔCT** | **s.e.m** | **FC** | **p** | **ΔCT** | **s.e.m** | **ΔΔCT** | **s.e.m** | **FC** | **p** | **ΔCT** | **s.e.m** | **ΔΔCT** | **s.e.m** | **FC** | **p** |  |
|  |  | **ABCG8** | |  |  |  |  | **CCL22** |  |  |  |  |  | **CXCL10** | |  |  |  |  |  |
| **HT29/M0** | L3 | n/e |  |  |  |  |  | 27.17 | 0.42 | 3.91 | 0.94 | -15 | 0.001 | 23.31 | 0.22 | 0.87 | 0.54 | -1.8 | 0.129 |  |
|  | L4 | n/e |  |  |  |  |  | 26.79 | 0.42 | -1.64 | 0.94 | 3.1 | 0.108 | 23.32 | 0.22 | 0.59 | 0.54 | -1.5 | 0.296 |  |
| *Overall p value* | |  |  |  |  |  |  |  |  |  |  |  | ***0.003*** |  |  |  |  |  | *0.187* |  |
| **SW480/M0** | L3 | n/e |  |  |  |  |  | 25.5 | 0.47 | 1.37 | 1.05 | -2.6 | 0.242 | n/e |  |  |  |  |  |  |
|  | L4 | n/e |  |  |  |  |  | 27.74 | 0.94 | -0.31 | -0.94 | 1.24 | 0.242 | n/e |  |  |  |  |  |  |
| *Overall p value* | |  |  |  |  |  |  |  |  |  |  |  | *0.242* |  |  |  |  |  |  |  |
| **HT29/M2** | L3 | n/e |  |  |  |  |  | 25.56 | 0.52 | 0.39 | 1.17 | -1.3 | 0.741 | 19.22 | 0.18 | 0.56 | 0.44 | -1.5 | 0.214 |  |
|  | L4 | n/e |  |  |  |  |  | 27.02 | 0.47 | -0.58 | 1.15 | 1.5 | 0.624 | 19.46 | 0.18 | 0.14 | 0.44 | -1.1 | 0.758 |  |
| *Overall p value* | |  |  |  |  |  |  |  |  |  |  |  | *0.835* |  |  |  |  |  | *0.432* |  |
| **SW480/M2** | L3 | 23.71 | 0.62 | -0.54 | 1.39 | 1.5 | 0.702 | 26.63 | 0.67 | 3.37 | 1.65 | -10.3 | 0.059 | 23.31 | 0.22 | 0.87 | 0.54 | -1.8 | 0.129 |  |
|  | L4 | 24.37 | 0.62 | 4.15 | 1.39 | -17.8 | 0.011 | 27.39 | 0.67 | -1.05 | 1.65 | 2.1 | 0.536 | 23.32 | 0.22 | 0.59 | 0.54 | -1.5 | 0.296 |  |
| *Overall p value* | |  |  |  |  |  | ***0.034*** |  |  |  |  |  | *0.135* |  |  |  |  |  | *0.187* |  |
|  |  | **GATA5** | |  |  |  |  | **IL10** |  |  |  |  |  | **IL1β** | |  |  |  |  |  |
| **HT29/M0** | L3 | 15.11 | 0.33 | -1.65 | 0.67 | 3.1 | 0.03 | n/e |  |  |  |  |  | n/e |  |  |  |  |  |  |
|  | L4 | 14.33 | 0.26 | -1.49 | 0.63 | 2.8 | 0.037 | n/e |  |  |  |  |  | n/e |  |  |  |  |  |  |
| *Overall p value* | |  |  |  |  |  | ***0.017*** |  |  |  |  |  |  |  |  |  |  |  |  |  |
| **SW480/M0** | L3 | n/e |  |  |  |  |  | 17.54 | 0.16 | 0.33 | 0.38 | -1.3 | 0.405 | n/e |  |  |  |  |  |  |
|  | L4 | n/e |  |  |  |  |  | 21.54 | 0.16 | 1.1 | 0.38 | -2.2 | 0.011 | n/e |  |  |  |  |  |  |
| *Overall p value* | |  |  |  |  |  |  |  |  |  |  |  | ***0.028*** |  |  |  |  |  |  |  |
| **HT29/M2** | L3 | 28.53 | 0.13 | 3.08 | 0.26 | -8.5 | <0.001 | n/e |  |  |  |  |  | 28.55 | 0.52 | 1.23 | 1.05 | -2.3 | 0.266 |  |
|  | L4 | 24.31 | 0.11 | -2.32 | 0.26 | 5 | <0.001 | n/e |  |  |  |  |  | 26.42 | 0.45 | 0.19 | 1.01 | -1.1 | 0.853 |  |
| *Overall p value* | |  |  |  |  |  | ***<0.001*** |  |  |  |  |  |  |  |  |  |  |  | *0.514* |  |
| **SW480/M2** | L3 | n/e |  |  |  |  |  | n/e |  |  |  |  |  | 24.47 | 0.34 | -2.11 | 0.84 | 4.3 | 0.023 |  |
|  | L4 | n/e |  |  |  |  |  | n/e |  |  |  |  |  | 24.95 | 0.34 | -1.71 | 0.84 | 3.3 | 0.058 |  |
| *Overall p value* | |  |  |  |  |  |  |  |  |  |  |  |  |  |  |  |  |  | ***0.018*** |  |
|  |  | **IL8** | |  |  |  |  | **LCT** |  |  |  |  |  | **NFκB** | |  |  |  |  |  |
| **HT29/M0** | L3 | 18.37 | 0.07 | 0.16 | 0.17 | -1.1 | 0.388 | n/e |  |  |  |  |  | n/e |  |  |  |  |  |  |
|  | L4 | 20.32 | 0.07 | 0.25 | 0.17 | -1.2 | 0.169 | n/e |  |  |  |  |  | n/e |  |  |  |  |  |  |
| *Overall p value* | |  |  |  |  |  | *0.268* |  |  |  |  |  |  |  |  |  |  |  |  |  |
| **SW480/M0** | L3 | 18.45 | 0.15 | -0.05 | 0.38 | 1 | 0.899 | n/e |  |  |  |  |  | 15.54 | 0.11 | 0.01 | 0.26 | -1 | 0.976 |  |
|  | L4 | 20.66 | 0.15 | 1.04 | 0.38 | -2.1 | 0.014 | 25.13 | 0.63 | -0.99 | 1.27 | 2 | 0.48 | 17.32 | 0.11 | 0.23 | 0.26 | -1.2 | 0.392 |  |
| *Overall p value* | |  |  |  |  |  | *0.044* |  |  |  |  |  | 0.48 |  |  |  |  |  | *0.685* |  |
| **HT29/M2** | L3 | 19.61 | 0.18 | -0.32 | 0.41 | 1.2 | 0.458 |  |  |  |  |  |  | 17.25 | 0.12 | 0.62 | 0.28 | -1.5 | 0.041 |  |
|  | L4 | 19.93 | 0.17 | -0.13 | 0.41 | 1.1 | 0.752 | n/e |  |  |  |  |  | 17.92 | 0.11 | 0.38 | 0.27 | -1.3 | 0.187 |  |
| *Overall p value* | |  |  |  |  |  | *0.715* | n/e |  |  |  |  |  |  |  |  |  |  | *0.058* |  |
| **SW480/M2** | L3 | 18.37 | 0.07 | 0.16 | 0.17 | -1.1 | 0.388 | 12.11 | 0.37 | -4.04 | 0.82 | 16.5 | <0.001 | 16.7 | 0.06 | 0.23 | 0.15 | -1.2 | 0.146 |  |
|  | L4 | 20.32 | 0.07 | 0.25 | 0.17 | -1.2 | 0.169 | 12.51 | 0.37 | 7.47 | 0.82 | -177.8 | <0.001 | 17.51 | 0.06 | 0.23 | 0.15 | -1.2 | 0.153 |  |
| *Overall p value* | |  |  |  |  |  | *0.268* |  |  |  |  |  | ***<0.001*** |  |  |  |  |  | *0.133* |  |
|  |  | **ΔCT** | **s.e.m** | **ΔΔCT** | **s.e.m** | **FC** | **p** | **ΔCT** | **s.e.m** | **ΔΔCT** | **s.e.m** | **FC** | **p** | **ΔCT** | **s.e.m** | **ΔΔCT** | **s.e.m** | **FC** | **p** |  |
|  |  | **PLIN4** | |  |  |  |  | **PPARα** | |  |  |  |  | **PPARγ** | |  |  |  |  |  |
| **HT29/M0** | L3 | 19.93 | 0.37 | 0.29 | 0.9 | -1.2 | 0.752 | 20.07 | 0.1 | 0.45 | 0.24 | -1.4 | 0.077 | 15.99 | 0.1 | -0.18 | 0.24 | 1.1 | 0.462 |  |
|  | L4 | 19.34 | 0.37 | 0.13 | 0.9 | -1.1 | 0.886 | 19.64 | 0.1 | -0.24 | 0.24 | 1.2 | 0.34 | 16.4 | 0.1 | -0.54 | 0.24 | 1.5 | 0.038 |  |
| *Overall p value* | |  |  |  |  |  | *0.94* |  |  |  |  |  | *0.135* |  |  |  |  |  | *0.087* |  |
| **SW480/M0** | L3 | 23.88 | 0.28 | 0.02 | 0.64 | -1 | 0.977 | 21.93 | 0.2 | -0.01 | 0.48 | 1 | 0.987 | 18.28 | 0.13 | 0.1 | 0.31 | -1.1 | 0.751 |  |
|  | L4 | 23.22 | 0.25 | -0.51 | 0.62 | 1.4 | 0.424 | 21.93 | 0.2 | -0.01 | 0.48 | 1 | 0.987 | 21.79 | 0.13 | 0.2 | 0.31 | -1.1 | 0.53 |  |
| *Overall p value* | |  |  |  |  |  | *0.717* |  |  |  |  |  | *1* |  |  |  |  |  | *0.776* |  |
| **HT29/M2** | L3 | 20.98 | 0.15 | 0.31 | 0.37 | -1.2 | 0.419 | 20.26 | 0.17 | -0.21 | 0.37 | 1.2 | 0.578 | 16.83 | 0.24 | -0.93 | 0.59 | 1.9 | 0.136 |  |
|  | L4 | 20.06 | 0.15 | -0.27 | 0.37 | 1.2 | 0.481 | 19.75 | 0.15 | -0.43 | 0.36 | 1.3 | 0.262 | 16.98 | 0.27 | 0.41 | 0.6 | -1.3 | 0.512 |  |
| *Overall p value* | |  |  |  |  |  | *0.558* |  |  |  |  |  | *0.451* |  |  |  |  |  | *0.262* |  |
| **SW480/M2** | L3 | 23.9 | 0.23 | -0.52 | 0.57 | 1.4 | 0.377 | 20.07 | 0.1 | 0.45 | 0.24 | -1.4 | 0.077 | 19.23 | 0.14 | -0.01 | 0.34 | 1 | 0.966 |  |
|  | L4 | 23.34 | 0.23 | 5.51 | 0.57 | -45.6 | <0.001 | 19.64 | 0.1 | -0.24 | 0.24 | 1.2 | 0.34 | 19.82 | 0.14 | 1.17 | 0.34 | -2.3 | 0.004 |  |
| *Overall p value* | |  |  |  |  |  | ***<0.001*** |  |  |  |  |  | *0.135* |  |  |  |  |  | ***0.012*** |  |
|  |  | **SERPINE** | |  |  |  |  | **TNFα** | |  |  |  |  |  |  |  |  |  |  |  |
| **HT29/M0** | L3 | 20.58 | 0.16 | -0.28 | 0.39 | 1.2 | 0.476 | 25.87 | 0.34 | 0.32 | 0.76 | -1.3 | 0.679 |  |  |  |  |  |  |  |
|  | L4 | 20.74 | 0.16 | -0.38 | 0.39 | 1.3 | 0.348 | 27.03 | 0.3 | 0.03 | 0.75 | -1 | 0.972 |  |  |  |  |  |  |  |
| *Overall p value* | |  |  |  |  |  | *0.496* |  |  |  |  |  | *0.914* |  |  |  |  |  |  |  |
| **SW480/M0** | L3 | 15.58 | 0.16 | 0 | 0.4 | 1 | 0.995 | n/e |  |  |  |  |  |  |  |  |  |  |  |  |
|  | L4 | 17.74 | 0.16 | 0.52 | 0.4 | -1.4 | 0.208 | 27.45 | 0.46 | -0.6 | 1.02 | 1.5 | 0.578 |  |  |  |  |  |  |  |
| *Overall p value* | |  |  |  |  |  | *0.442* |  |  |  |  |  | *0.578* |  |  |  |  |  |  |  |
| **HT29/M2** | L3 | 22.97 | 0.16 | 0.03 | 0.4 | -1 | 0.937 | 25.95 | 0.47 | 0.48 | 1.06 | -1.4 | 0.656 |  |  |  |  |  |  |  |
|  | L4 | 22.18 | 0.16 | 0.76 | 0.4 | -1.7 | 0.079 | 26.14 | 0.55 | -0.17 | 1.09 | 1.1 | 0.877 |  |  |  |  |  |  |  |
| *Overall p value* | |  |  |  |  |  | *0.202* |  |  |  |  |  | *0.89* |  |  |  |  |  |  |  |
| **SW480/M2** | L3 | 17.3 | 0.13 | -0.38 | 0.32 | 1.3 | 0.257 | 27.82 | 0.87 | 2.73 | 2.13 | -6.6 | 0.217 |  |  |  |  |  |  |  |
|  | L4 | 16.33 | 0.13 | 0.53 | 0.32 | -1.4 | 0.123 | 27.09 | 0.87 | 1.58 | 2.13 | -3 | 0.467 |  |  |  |  |  |  |  |
| *Overall p value* | |  |  |  |  |  | *0.166* |  |  |  |  |  | *0.356* |  |  |  |  |  |  |  |

**Supplementary Table 9. Cancer Cell Line Gene Expression Following Leptin Treatment.** Cancer cell line gene expression following leptin treatment with M0 or M2 macrophage co-culture. s.e.m: standard error of the mean, FC: fold change, L3:6-hour treatment, L4: 18-hour treatment. n/e: not expressed. Boldface reflects overall significant change in gene expression (p<0.05). *CD206 and IL6 not expressed in all experimental conditions (data not shown)*.

|  | | | | | | | | | | | | | | | | | | | |  |
| --- | --- | --- | --- | --- | --- | --- | --- | --- | --- | --- | --- | --- | --- | --- | --- | --- | --- | --- | --- | --- |
| Cell lines/  Treatment length | | **ΔCT** | **s.e.m** | **ΔΔCT** | **s.e.m** | **FC** | **p** | **ΔCT** | **s.e.m** | **ΔΔCT** | **s.e.m** | **FC** | **p** | **ΔCT** | **s.e.m** | **ΔΔCT** | **s.e.m** | **FC** | **p** |  |
|  |  | **ABCG8** | |  |  |  |  | **CCL22** |  |  |  |  |  | **CXCL10** | |  |  |  |  |  |
| **HT29/M0** | L3 | n/e |  |  |  |  |  | 23.36 | 0.31 | 0.23 | 0.76 | -1.2 | 0.773 | 24.14 | 1.27 | 0.43 | 3.11 | -1.4 | 0.892 |  |
|  | L4**^a^** | **-** | **-** | **-** | **-** | **-** | **-** | **-** | **-** | **-** | **-** | **-** | **-** | **-** | **-** | **-** | **-** | **-** |  |  |
| *Overall p value* | |  |  |  |  |  |  |  |  |  |  |  | *0.773* |  |  |  |  |  | *0.892* |  |
| **SW480/M0** | L3 | 27.43 | 0.7 | -1.85 | 1.72 | 3.6 | 0.297 | 26.19 | 0.67 | -2.44 | 1.64 | 5.4 | 0.156 | n/e |  |  |  |  |  |  |
|  | L4 | 28.37 | 0.7 | 0.2 | 1.72 | -1.1 | 0.908 | 25.05 | 0.67 | -3.98 | 1.64 | 15.8 | 0.027 | n/e |  |  |  |  |  |  |
| *Overall p value* | |  |  |  |  |  | *0.567* |  |  |  |  |  | ***0.038*** |  |  |  |  |  |  |  |
| **HT29/M2** | L3 | n/e |  |  |  |  |  | 26.23 | 0.71 | -1.37 | 1.42 | 2.6 | 0.354 | 18.98 | 0.47 | 1.93 | 1.04 | -3.8 | 0.084 |  |
|  | L4 | n/e |  |  |  |  |  | 28.44 | 0.55 | 2.54 | 1.35 | -5.8 | 0.084 | 19.17 | 0.42 | 2.36 | 1.02 | -5.1 | 0.036 |  |
| *Overall p value* | |  |  |  |  |  |  |  |  |  |  |  | *0.149* |  |  |  |  |  | ***0.033*** |  |
| **SW480/M2** | L3 | n/e |  |  |  |  |  | n/e |  |  |  |  |  | n/e |  |  |  |  |  |  |
|  | L4 **^a^** | **-** | **-** | **-** | **-** | **-** | **-** | **-** | **-** | **-** | **-** | **-** | **-** | **-** | **-** | **-** | **-** | **-** |  |  |
| *Overall p value* | |  |  |  |  |  |  |  |  |  |  |  |  |  |  |  |  |  |  |  |
|  |  | **GATA5** | |  |  |  |  | **IL10** |  |  |  |  |  | **IL1β** | |  |  |  |  |  |
| **HT29/M0** | L3 | 26.87 | 0.59 | -2.3 | 1.46 | 4.9 | 0.153 | n/e |  |  |  |  |  | 21.23 | 1.13 | -1.17 | 2.78 | 2.3 | 0.683 |  |
|  | L4 **^a^** | - | - | - | - | - | - | - | - | - | - | - | - | - | - | - | - | - | - |  |
| *Overall p value* | |  |  |  |  |  | *0.153* |  |  |  |  |  |  |  |  |  |  |  | *0.683* |  |
| **SW480/M0** | L3 | n/e |  |  |  |  |  | 21.91 | 0.47 | 3.34 | 1.15 | -10.1 | 0.01 | n/e |  |  |  |  |  |  |
|  | L4 | n/e |  |  |  |  |  | 25.0 | 0.47 | 4.6 | 1.15 | -24.2 | 0.001 | 25.86 | 1 | -3.18 | 2.45 | 9 | 0.231 |  |
| *Overall p value* | |  |  |  |  |  |  |  |  |  |  |  | ***0.001*** |  |  |  |  |  | *0.231* |  |
| **HT29/M2** | L3 | n/e |  |  |  |  |  | n/e |  |  |  |  |  | 25.97 | 0.84 | -1.84 | 2.06 | 3.6 | 0.385 |  |
|  | L4 | n/e |  |  |  |  |  | n/e |  |  |  |  |  | 26.63 | 0.94 | -0.36 | 2.1 | 1.3 | 0.868 |  |
| *Overall p value* | |  |  |  |  |  |  |  |  |  |  |  |  |  |  |  |  |  | *0.667* |  |
| **SW480/M2** | L3 | n/e |  |  |  |  |  | 23.94 | 0.41 | 3.32 | 0.92 | -10 | 0.011 | n/e |  |  |  |  |  |  |
|  | L4 **^a^** | - | - | - | - | - | - | - | - | - | - | - | - | - | - | - | - | - | - |  |
| *Overall p value* | |  |  |  |  |  |  |  |  |  |  |  | ***0.011*** |  |  |  |  |  |  |  |
|  |  | **IL6** | |  |  |  |  | **IL8** |  |  |  |  |  | **LCT** | |  |  |  |  |  |
| **HT29/M0** | L3 | n/e |  |  |  |  |  | 17.48 | 0.08 | 0.37 | 0.2 | -1.3 | 0.093 | n/e |  |  |  |  |  |  |
|  | L4 **^a^** | - | - | - | - | - | - | - | - | - | - | - | - | - | - | - | - | - | - |  |
| *Overall p value* | |  |  |  |  |  |  |  |  |  |  |  | *0.093* |  |  |  |  |  |  |  |
| **SW480/M0** | L3 | n/e |  |  |  |  |  | 20.7 | 0.5 | -0.15 | 1.22 | 1.1 | 0.906 | 24.46 | 0.97 | -0.62 | 2.37 | 1.5 | 0.797 |  |
|  | L4 | 26.12 | 0.38 | -2.24 | 0.94 | 4.7 | 0.044 | 22.5 | 0.5 | 2.99 | 1.22 | -7.9 | 0.026 | 26.92 | 0.97 | 1.22 | 2.37 | -2.3 | 0.613 |  |
| *Overall p value* | |  |  |  |  |  | ***0.044*** |  |  |  |  |  | *0.077* |  |  |  |  |  | *0.848* |  |
| **HT29/M2** | L3 | n/e |  |  |  |  |  | 20.66 | 1.28 | 1.14 | 2.85 | -2.2 | 0.695 | n/e |  |  |  |  |  |  |
|  | L4 | n/e |  |  |  |  |  | 23.09 | 1.14 | 2.22 | 2.79 | -4.7 | 0.44 | n/e |  |  |  |  |  |  |
| *Overall p value* | |  |  |  |  |  |  |  |  |  |  |  | *0.681* |  |  |  |  |  |  |  |
| **SW480/M2** | L3 | n/e |  |  |  |  |  | 21.4 | 0.26 | 0.89 | 0.64 | -1.9 | 0.204 | 24.95 | 0.47 | -2.44 | 1.05 | 5.4 | 0.06 |  |
|  | L4 **^a^** | - | - | - | - | - | - | - | - | - | - | - | - | - | - | - | - | - | - |  |
| *Overall p value* | |  |  |  |  |  |  |  |  |  |  |  | *0.204* |  |  |  |  |  | *0.06* |  |
|  |  | **ΔCT** | **s.e.m** | **ΔΔCT** | **s.e.m** | **FC** | **p** | **ΔCT** | **s.e.m** | **ΔΔCT** | **s.e.m** | **FC** | **p** | **ΔCT** | **s.e.m** | **ΔΔCT** | **s.e.m** | **FC** | **p** |  |
|  |  | **CD206** | |  |  |  |  | **NFkB** | |  |  |  |  | **PLIN4** | |  |  |  |  |  |
| **HT29/M0** | L3 | 25.24 | 0.65 | -2.17 | 1.59 | 4.5 | 0.207 | 18.48 | 0.28 | 1.99 | 0.69 | -4 | 0.021 | 16.97 | 0.25 | -0.52 | 0.62 | 1.4 | 0.428 |  |
|  | L4 **^a^** | - | - | - | - | - | - | - | - | - | - | - | - | - | - | - | - | - | - |  |
| *Overall p value* | |  |  |  |  |  | *0.207* |  |  |  |  |  | ***0.021*** |  |  |  |  |  | *0.428* |  |
| **SW480/M0** | L3 | 26.54 | 0.48 | -1 | 1.17 | 2 | 0.406 | 16.69 | 0.2 | 0.41 | 0.48 | -1.3 | 0.408 | 20.75 | 0.15 | -3.43 | 0.36 | 10.8 | <0.001 |  |
|  | L4 | 25.53 | 0.48 | -2.63 | 1.17 | 6.2 | 0.039 | 18.58 | 0.2 | 1.53 | 0.48 | -2.9 | 0.006 | 19.64 | 0.15 | -5 | 0.36 | 32.1 | <0.001 |  |
| *Overall p value* | |  |  |  |  |  | *0.085* |  |  |  |  |  | ***0.016*** |  |  |  |  |  | ***<0.001*** |  |
| **HT29/M2** | L3 | 20.63 | 0.16 | 1.51 | 0.39 | -2.8 | 0.001 | 17.3 | 0.1 | 1 | 0.25 | -2 | 0.001 | 18.81 | 0.54 | -2.84 | 1.32 | 7.1 | 0.047 |  |
|  | L4 | 20.48 | 0.16 | 2.06 | 0.39 | -4.2 | <0.001 | 17.27 | 0.1 | 1.31 | 0.25 | -2.5 | <0.001 | 17.93 | 0.54 | -2.75 | 1.32 | 6.7 | 0.054 |  |
| *Overall p value* | |  |  |  |  |  | ***<0.001*** |  |  |  |  |  | ***<0.001*** |  |  |  |  |  | ***0.028*** |  |
| **SW480/M2** | L3 | 26.52 | 0.13 | -2.53 | 0.33 | 5.8 | <0.001 | 18.12 | 0.16 | 0.59 | 0.39 | -1.5 | 0.173 | 23.66 | 0.29 | -1.15 | 0.64 | 2.2 | 0.124 |  |
|  | L4 **^a^** | - | - | - | - | - | - | - | - | - | - | - | - | - | - | - | - | - | - |  |
| *Overall p value* | |  |  |  |  |  | ***<0.001*** |  |  |  |  |  | *0.173* |  |  |  |  |  | *0.124* |  |
|  |  | **PPARα** | |  |  |  |  | **PPARγ** | |  |  |  |  | **SERPINE** | |  |  |  |  |  |
| **HT29/M0** | L3 | 20.38 | 0.3 | 0.32 | 0.73 | -1.2 | 0.676 | 18.72 | 0.28 | 2.25 | 0.68 | -4.7 | 0.011 | 20.28 | 0.34 | -0.7 | 0.82 | 1.6 | 0.422 |  |
|  | L4 **^a^** | - | - | - | - | - | - | - | - | - | - | - | - | - | - | - | - | - | - |  |
| *Overall p value* | |  |  |  |  |  | *0.676* |  |  |  |  |  | ***0.011*** |  |  |  |  |  | *0.422* |  |
| **SW480/M0** | L3 | 22.38 | 0.18 | -0.51 | 0.44 | 1.4 | 0.258 | 19.32 | 0.23 | 0.04 | 0.56 | -1 | 0.941 | 15.48 | 0.13 | -1.63 | 0.32 | 3.1 | <0.001 |  |
|  | L4 | 21.85 | 0.18 | 0.43 | 0.44 | -1.3 | 0.344 | 22.93 | 0.23 | 1.66 | 0.56 | -3.2 | 0.009 | 13.6 | 0.13 | -1.77 | 0.32 | 3.4 | <0.001 |  |
| *Overall p value* | |  |  |  |  |  | *0.338* |  |  |  |  |  | ***0.03*** |  |  |  |  |  | ***<0.001*** |  |
| **HT29/M2** | L3 | 18.7 | 0.22 | -0.74 | 0.5 | 1.7 | 0.163 | 20.18 | 0.16 | 1.22 | 0.38 | -2.3 | 0.005 | 19.99 | 0.42 | -0.93 | 1.04 | 1.9 | 0.383 |  |
|  | L4 | 17.76 | 0.2 | -0.49 | 0.49 | 1.4 | 0.335 | 18.64 | 0.16 | 0.17 | 0.38 | -1.1 | 0.659 | 19.01 | 0.42 | -0.93 | 1.04 | 1.9 | 0.38 |  |
| *Overall p value* | |  |  |  |  |  | *0.24* |  |  |  |  |  | ***0.018*** |  |  |  |  |  | *0.462* |  |
| **SW480/M2** | L3 | 23.86 | 0.19 | 0.04 | 0.47 | -1 | 0.941 | 20.61 | 0.16 | 0.4 | 0.39 | -1.3 | 0.328 | 17.53 | 0.19 | -0.98 | 0.47 | 2 | 0.069 |  |
|  | L4 **^a^** | - | - | - | - | - | - | - | - | - | - | - | - | - | - | - | - | - | - |  |
| *Overall p value* | |  |  |  |  |  | *0.941* |  |  |  |  |  | *0.328* |  |  |  |  |  | *0.069* |  |
|  |  | **TNFα** | |  |  |  |  |  |  |  |  |  |  |  |  |  |  |  |  |  |
| **HT29/M0** | L3 | 23.08 | 1.14 | -0.1 | 2.8 | 1.1 | 0.973 |  |  |  |  |  |  |  |  |  |  |  |  |  |
|  | L4 **^a^** | - | - | - | - | - | - |  |  |  |  |  |  |  |  |  |  |  |  |  |
| *Overall p value* | |  |  |  |  |  | *0.973* |  |  |  |  |  |  |  |  |  |  |  |  |  |
| **SW480/M0** | L3 | 25.05 | 0.77 | -3.57 | 1.9 | 11.8 | 0.078 |  |  |  |  |  |  |  |  |  |  |  |  |  |
|  | L4 | 22.94 | 0.77 | -4.26 | 1.9 | 19.2 | 0.039 |  |  |  |  |  |  |  |  |  |  |  |  |  |
| *Overall p value* | |  |  |  |  |  | ***0.032*** |  |  |  |  |  |  |  |  |  |  |  |  |  |
| **HT29/M2** | L3 | 22.95 | 0.72 | 3.68 | 1.6 | -12.8 | 0.038 |  |  |  |  |  |  |  |  |  |  |  |  |  |
|  | L4 | 24.39 | 0.64 | 7.05 | 1.57 | -132.7 | 0.001 |  |  |  |  |  |  |  |  |  |  |  |  |  |
| *Overall p value* | |  |  |  |  |  | ***0.001*** |  |  |  |  |  |  |  |  |  |  |  |  |  |
| **SW480/M2** | L3 | 26.89 | 0.4 | 0.42 | 0.97 | -1.3 | 0.677 |  |  |  |  |  |  |  |  |  |  |  |  |  |
|  | L4 **^a^** | - | - | - | - | - | - |  |  |  |  |  |  |  |  |  |  |  |  |  |
| *Overall p value* | |  |  |  |  |  | *0.677* |  |  |  |  |  |  |  |  |  |  |  |  |  |

**Supplementary Table 10. Cancer Cell Line Gene Expression Following 4-Octyl Itaconate Treatment.** Cancer cell line gene expression following 4-octyl itaconate treatment with M0 or M2 macrophage co-culture. s.e.m: standard error of the mean, FC: fold change, L3:6-hour treatment, L4: 18-hour treatment. n/e: not expressed. Boldface reflects overall significant change in gene expression (p<0.05). ^a^ 4OI caused macrophage death following 18 hours treatment in M0/HT29 and M2/SW480 therefore gene expression not measured.

|  | | | | | | | | | | | | | | | | | | | |  |
| --- | --- | --- | --- | --- | --- | --- | --- | --- | --- | --- | --- | --- | --- | --- | --- | --- | --- | --- | --- | --- |
| Cell lines/  Treatment length | | **ΔCT** | **s.e.m** | **ΔΔCT** | **s.e.m** | **FC** | **p** | **ΔCT** | **s.e.m** | **ΔΔCT** | **s.e.m** | **FC** | **p** | **ΔCT** | **s.e.m** | **ΔΔCT** | **s.e.m** | **FC** | **p** |  |
|  |  | **ABCG8** | |  |  |  |  | **CCL22** |  |  |  |  |  | **CXCL10** | |  |  |  |  |  |
| **HT29/M0** | L3 | n/e |  |  |  |  |  | 24.51 | 0.72 | -2.89 | 1.77 | 7.4 | 0.14 | 22.31 | 0.45 | -3.53 | 1.11 | 11.5 | 0.013 |  |
|  | L4 | n/e |  |  |  |  |  | 30.45 | 0.42 | 0.52 | 0.42 | 0.70 | 0.14 |  |  |  |  |  |  |  |
| *Overall p value* | |  |  |  |  |  |  |  |  |  |  |  | *0.14* |  |  |  |  |  | *0.013* |  |
| **SW480/M0** | L3 | 28.62 | 0.14 | 0.49 | 0.35 | -1.4 | 0.194 | n/e |  |  |  |  |  |  |  |  |  |  |  |  |
|  | L4 | n/e |  |  |  |  |  | 21.18 | 0.21 | 0.9 | 0.52 | -1.9 | 0.12 |  |  |  |  |  |  |  |
| *Overall p value* | |  |  |  |  |  | *0.194* |  |  |  |  |  | *0.12* |  |  |  |  |  |  |  |
| **HT29/M2** | L3 | n/e |  |  |  |  |  | 28.42 | 0.6 | -1.71 | 1.33 | 3.3 | 0.221 | 26.57 | 0.66 | 1.9 | 1.48 | -3.7 | 0.221 |  |
|  | L4 | n/e |  |  |  |  |  | 27.69 | 0.53 | -0.1 | 1.31 | 1.1 | 0.937 | 27.5 | 0.59 | 2.64 | 1.45 | -6.2 | 0.091 |  |
| *Overall p value* | |  |  |  |  |  |  |  |  |  |  |  | *0.459* |  |  |  |  |  | *0.121* |  |
| **SW480/M2** | L3 | n/e |  |  |  |  |  | n/e |  |  |  |  |  | n/e |  |  |  |  |  |  |
|  | L4 | n/e |  |  |  |  |  | n/e |  |  |  |  |  | n/e |  |  |  |  |  |  |
| *Overall p value* | |  |  |  |  |  |  |  |  |  |  |  |  |  |  |  |  |  |  |  |
|  |  | **GATA5** | |  |  |  |  | **IL10** |  |  |  |  |  | **IL1β** | |  |  |  |  |  |
| **HT29/M0** | L3 | n/e |  |  |  |  |  | n/e |  |  |  |  |  | 22.25 | 0.56 | -1.22 | 1.37 | 2.3 | 0.389 |  |
|  | L4 | 26.99 | 0.65 | 1.83 | 1.45 | -3.6 | 0.254 | n/e |  |  |  |  |  | 25.9 | 0.63 | -2.25 | 1.4 | 4.8 | 0.13 |  |
| *Overall p value* | |  |  |  |  |  | *0.254* |  |  |  |  |  |  |  |  |  |  |  | *0.221* |  |
| **SW480/M0** | L3 | n/e |  |  |  |  |  | 18.88 | 0.15 | -0.14 | 0.36 | 1.1 | 0.703 | n/e |  |  |  |  |  |  |
|  | L4 | n/e |  |  |  |  |  | 21.49 | 0.15 | 0.77 | 0.36 | -1.7 | 0.048 | 27.61 | 0.35 | 0.97 | 0.85 | -2 | 0.284 |  |
| *Overall p value* | |  |  |  |  |  |  |  |  |  |  |  | *0.126* |  |  |  |  |  | *0.284* |  |
| **HT29/M2** | L3 | 24.87 | 0.52 | -1.33 | 1.17 | 2.5 | 0.274 | n/e |  |  |  |  |  | 25.7 | 0.45 | -0.08 | 1.1 | 1.1 | 0.94 |  |
|  | L4 | 27.14 | 0.47 | 3.36 | 1.14 | -10.3 | 0.011 | n/e |  |  |  |  |  | 25.55 | 0.45 | -0.34 | 1.1 | 1.3 | 0.762 |  |
| *Overall p value* | |  |  |  |  |  | ***0.023*** |  |  |  |  |  |  |  |  |  |  |  | *0.951* |  |
| **SW480/M2** | L3 | n/e |  |  |  |  |  | 21.01 | 0.09 | -0.21 | 0.21 | 1.2 | 0.346 | n/e |  |  |  |  |  |  |
|  | L4 | n/e |  |  |  |  |  | 21.22 | 0.09 | 0.24 | 0.21 | -1.2 | 0.28 | n/e |  |  |  |  |  |  |
| *Overall p value* | |  |  |  |  |  |  |  |  |  |  |  | *0.358* |  |  |  |  |  |  |  |
|  |  | **IL8** | |  |  |  |  | **LCT** |  |  |  |  |  | **CD206** | |  |  |  |  |  |
| **HT29/M0** | L3 | 17.16 | 0.26 | -0.17 | 0.63 | 1.1 | 0.787 | n/e |  |  |  |  |  | n/e |  |  |  |  |  |  |
|  | L4 | 22 | 0.26 | 0.21 | 0.63 | -1.2 | 0.745 | n/e |  |  |  |  |  | n/e |  |  |  |  |  |  |
| *Overall p value* | |  |  |  |  |  | *0.912* |  |  |  |  |  |  |  |  |  |  |  |  |  |
| **SW480/M0** | L3 | 20.5 | 0.17 | -0.14 | 0.41 | 1.1 | 0.732 | 25.63 | 0.88 | -2.03 | 1.77 | 4.1 | 0.278 | n/e |  |  |  |  |  |  |
|  | L4 | 20.91 | 0.17 | 1.29 | 0.41 | -2.4 | 0.006 | 27.4 | 0.77 | -0.49 | 1.71 | 1.4 | 0.783 | n/e |  |  |  |  |  |  |
| *Overall p value* | |  |  |  |  |  | ***0.02*** |  |  |  |  |  | *0.52* |  |  |  |  |  |  |  |
| **HT29/M2** | L3 | 21.28 | 0.76 | -0.42 | 1.86 | 1.3 | 0.824 | 27.39 | 0.19 | 0.64 | 0.42 | -1.6 | 0.175 | n/e |  |  |  |  |  |  |
|  | L4 | 21.27 | 0.76 | 0.67 | 1.86 | -1.6 | 0.723 | n/e |  |  |  |  |  | n/e |  |  |  |  |  |  |
| *Overall p value* | |  |  |  |  |  | *0.914* |  |  |  |  |  | *0.175* |  |  |  |  |  |  |  |
| **SW480/M2** | L3 | 20.85 | 0.12 | 0.56 | 0.31 | -1.5 | 0.088 | 26.3 | 0.41 | -0.53 | 0.92 | 1.4 | 0.571 | 29.32 | 0.26 | -0.61 | 0.64 | 1.5 | 0.354 |  |
|  | L4 | 20.54 | 0.12 | 0.6 | 0.31 | -1.5 | 0.068 | 25.89 | 0.41 | 0.93 | 0.92 | -1.9 | 0.332 | 29.74 | 0.26 | 0.44 | 0.64 | -1.4 | 0.501 |  |
| *Overall p value* | |  |  |  |  |  | *0.052* |  |  |  |  |  | *0.525* |  |  |  |  |  | *0.515* |  |
|  |  | **ΔCT** | **s.e.m** | **ΔΔCT** | **s.e.m** | **FC** | **p** | **ΔCT** | **s.e.m** | **ΔΔCT** | **s.e.m** | **FC** | **p** | **ΔCT** | **s.e.m** | **ΔΔCT** | **s.e.m** | **FC** | **p** |  |
|  |  | **NFkB** | |  |  |  |  | **PLIN4** | |  |  |  |  | **PPARα** | |  |  |  |  |  |
| **HT29/M0** | L3 | 17.3 | 0.33 | -3.79 | 0.8 | 13.8 | <0.001 | 19.53 | 0.33 | -4.62 | 0.81 | 24.6 | <0.001 | 19.39 | 0.27 | -4.35 | 0.66 | 20.4 | <0.001 |  |
|  | L4 | 18.33 | 0.33 | 0.71 | 0.8 | -1.6 | 0.39 | 20.85 | 0.33 | 0.57 | 0.81 | -1.5 | 0.492 | 20.15 | 0.27 | 0.34 | 0.66 | -1.3 | 0.612 |  |
| *Overall p value* | |  |  |  |  |  | ***0.001*** |  |  |  |  |  | ***<0.001*** |  |  |  |  |  | ***<0.001*** |  |
| **SW480/M0** | L3 | 16.59 | 0.07 | 0.27 | 0.18 | -1.2 | 0.162 | 24.13 | 0.42 | -0.13 | 1.03 | 1.1 | 0.899 | 22.05 | 0.18 | -0.17 | 0.43 | 1.1 | 0.692 |  |
|  | L4 | 17.61 | 0.07 | 0.47 | 0.18 | -1.4 | 0.021 | 26.17 | 0.47 | 1.44 | 1.05 | -2.7 | 0.191 | 22.36 | 0.18 | -0.37 | 0.43 | 1.3 | 0.409 |  |
| *Overall p value* | |  |  |  |  |  | ***0.032*** |  |  |  |  |  | *0.409* |  |  |  |  |  | *0.651* |  |
| **HT29/M2** | L3 | 16.64 | 0.72 | -0.01 | 1.77 | 1 | 0.997 | 19.93 | 0.35 | 0.16 | 0.86 | -1.1 | 0.851 | 19.81 | 0.74 | -0.38 | 1.81 | 1.3 | 0.838 |  |
|  | L4 | 17.03 | 0.72 | 0.21 | 1.77 | -1.2 | 0.908 | 19.16 | 0.35 | -0.69 | 0.86 | 1.6 | 0.433 | 19.09 | 0.74 | -0.18 | 1.81 | 1.1 | 0.922 |  |
| *Overall p value* | |  |  |  |  |  | *0.993* |  |  |  |  |  | *0.715* |  |  |  |  |  | *0.974* |  |
| **SW480/M2** | L3 | 16.99 | 0.09 | 0.02 | 0.21 | -1 | 0.915 | 24.35 | 0.21 | 0.27 | 0.51 | -1.2 | 0.607 | 23.91 | 0.11 | -0.24 | 0.28 | 1.2 | 0.406 |  |
|  | L4 | 17.18 | 0.09 | -0.08 | 0.21 | 1.1 | 0.714 | 24.59 | 0.21 | -0.97 | 0.51 | 2 | 0.077 | 22.54 | 0.11 | 0.04 | 0.28 | -1 | 0.896 |  |
| *Overall p value* | |  |  |  |  |  | *0.928* |  |  |  |  |  | *0.177* |  |  |  |  |  | *0.694* |  |
|  |  | **PPARγ** | |  |  |  |  | **SERPINE** | |  |  |  |  | **TNFα** | |  |  |  |  |  |
| **HT29/M0** | L3 | 16.53 | 0.32 | -2.28 | 0.79 | 4.9 | 0.011 | 21.36 | 0.29 | -3.39 | 0.72 | 10.5 | <0.001 | 25.7 | 0.64 | -2.02 | 1.58 | 4 | 0.222 |  |
|  | L4 | 17.47 | 0.32 | 0.55 | 0.79 | -1.5 | 0.497 | 23.32 | 0.29 | 0.45 | 0.72 | -1.4 | 0.541 | 28.92 | 0.72 | 3.43 | 1.61 | -10.8 | 0.051 |  |
| *Overall p value* | |  |  |  |  |  | ***0.03*** |  |  |  |  |  | ***0.001*** |  |  |  |  |  | *0.077* |  |
| **SW480/M0** | L3 | 19.31 | 0.15 | -0.09 | 0.36 | 1.1 | 0.811 | 16.88 | 0.21 | -0.3 | 0.51 | 1.2 | 0.559 | 29.22 | 0.62 | 2.42 | 1.38 | -5.4 | 0.101 |  |
|  | L4 | 21.19 | 0.15 | -0.21 | 0.36 | 1.2 | 0.567 | 18.32 | 0.21 | -0.84 | 0.51 | 1.8 | 0.118 | 28.65 | 0.55 | 1.73 | 1.35 | -3.3 | 0.22 |  |
| *Overall p value* | |  |  |  |  |  | *0.821* |  |  |  |  |  | *0.243* |  |  |  |  |  | *0.131* |  |
| **HT29/M2** | L3 | 16.99 | 0.7 | -0.07 | 1.7 | 1 | 0.969 | 22.66 | 0.59 | -0.15 | 1.45 | 1.1 | 0.917 | 28.84 | 0.42 | -1.21 | 0.94 | 2.3 | 0.222 |  |
|  | L4 | 16.62 | 0.7 | -0.06 | 1.7 | 1 | 0.972 | 21.9 | 0.59 | 0.02 | 1.45 | -1 | 0.988 | 28.43 | 0.42 | -1.28 | 0.94 | 2.4 | 0.199 |  |
| *Overall p value* | |  |  |  |  |  | *0.999* |  |  |  |  |  | *0.994* |  |  |  |  |  | *0.215* |  |
| **SW480/M2** | L3 | 20.82 | 0.11 | -0.35 | 0.28 | 1.3 | 0.234 | 18.79 | 0.11 | -0.34 | 0.27 | 1.3 | 0.223 | 27.59 | 0.67 | 1.62 | 1.64 | -3.1 | 0.338 |  |
|  | L4 | 20.69 | 0.11 | -0.58 | 0.28 | 1.5 | 0.054 | 17.69 | 0.11 | -0.54 | 0.27 | 1.5 | 0.064 | 28.45 | 0.67 | 2.18 | 1.64 | -4.5 | 0.202 |  |
| *Overall p value* | |  |  |  |  |  | *0.083* |  |  |  |  |  | *0.092* |  |  |  |  |  | *0.281* |  |

**Supplementary Table 11. Cancer Cell Line Gene Expression Following Dimethyl Itaconate Treatment.** Cancer cell line gene expression following dimethyl itaconate treatment with M0 or M2 macrophage co-culture. s.e.m: standard error of the mean, FC: fold change, L3:6-hour treatment, L4: 18-hour treatment. n/e: not expressed. Boldface reflects overall significant change in gene expression (p<0.05). *IL6 not expressed in all experimental conditions (data not shown)*.
