# Supplementary figures and images for: Itaconate and obesity-related hormones promote tumor progression – new insights on metabolic dysfunction in early-onset colon cancer
Source: Front Immunol. 2025 Jun 9;16:1572985. doi: 10.3389/fimmu.2025.1572985 (PMC12183228; doi:10.3389/fimmu.2025.1572985)

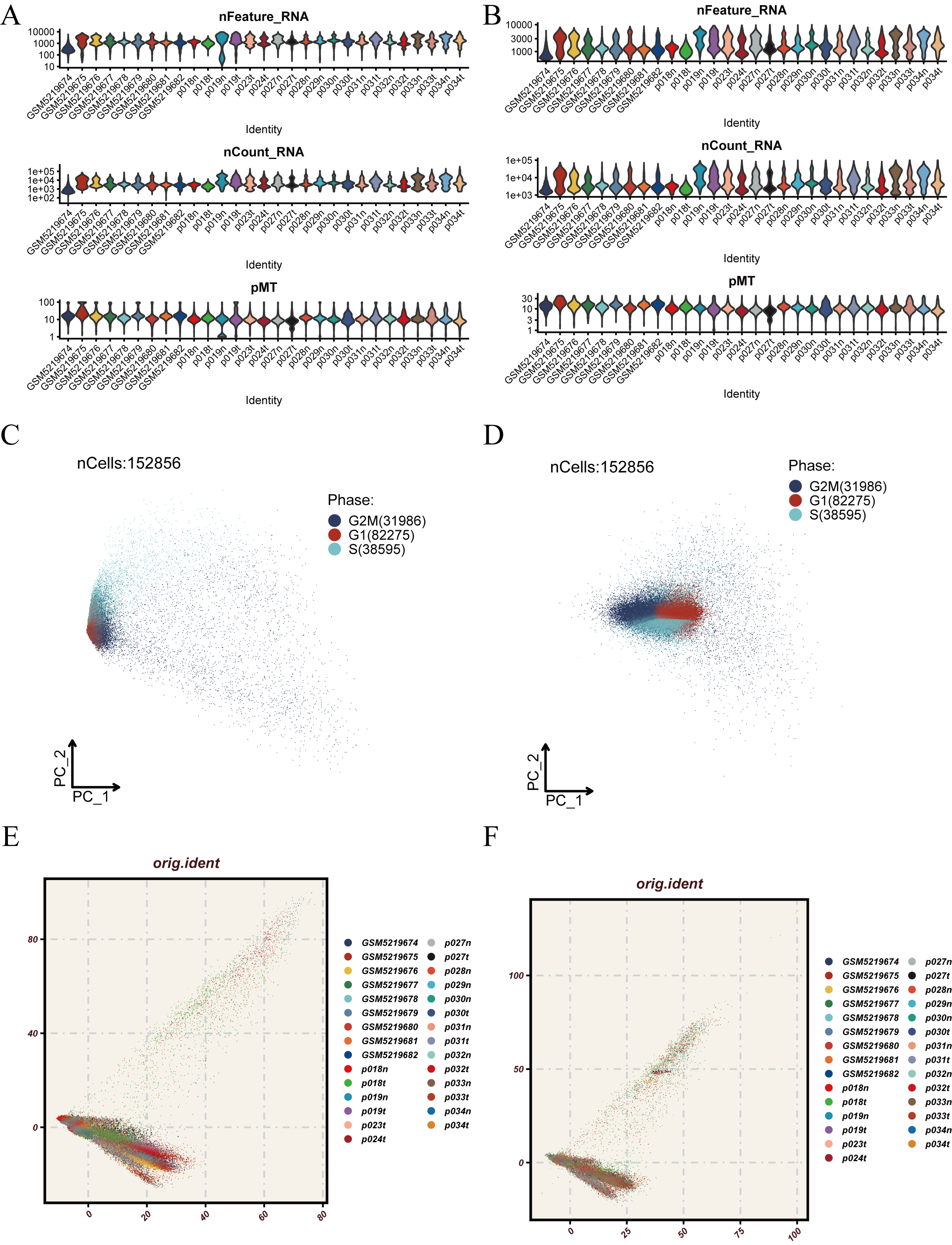

Supplement: Supplementary file 2 [file Image1.tif]

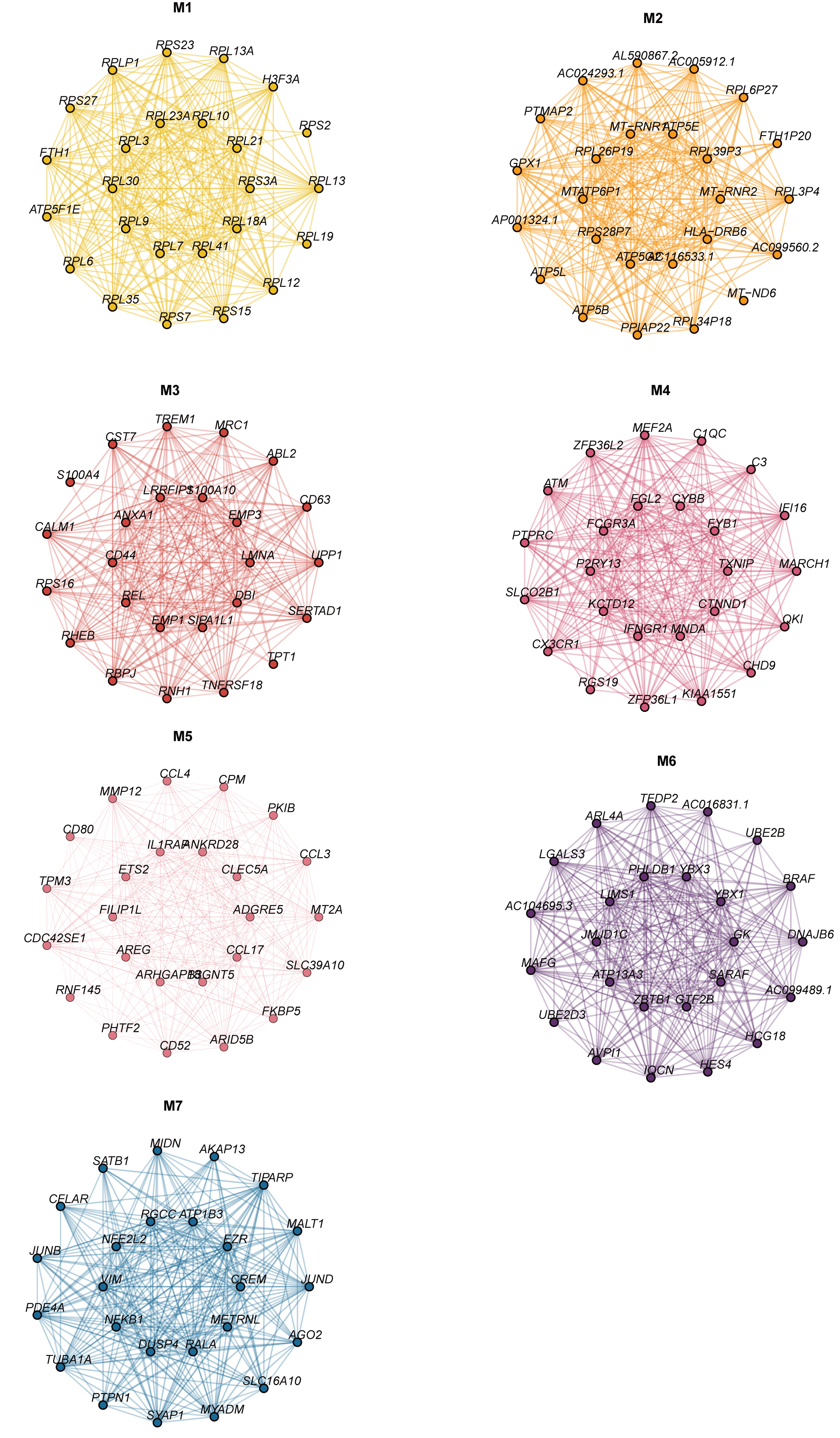

Supplement: Supplementary file 3 [file Image2.tif]

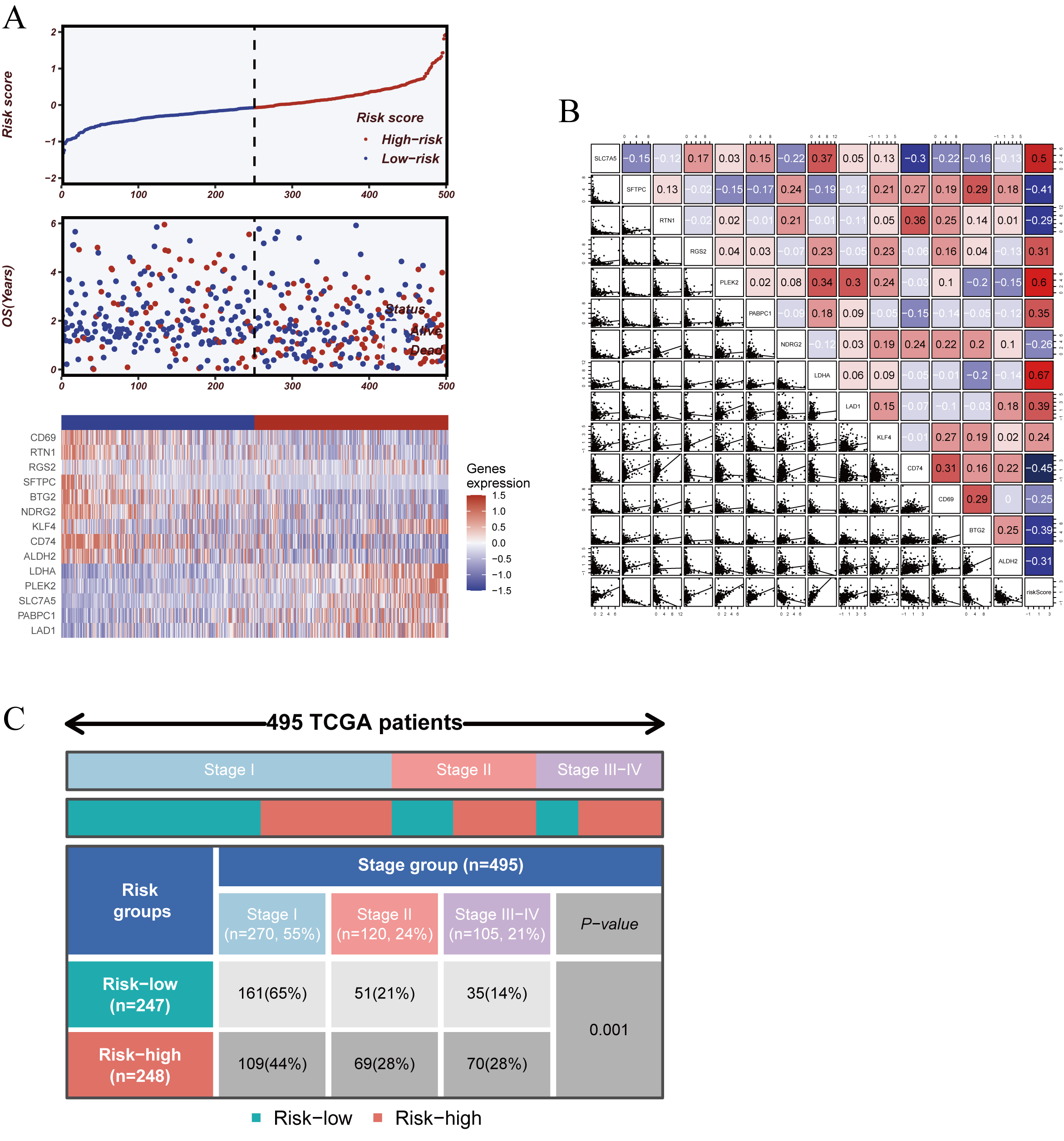

Supplement: Supplementary file 4 [file Image3.tif]
